# Anterior-temporal network hyperconnectivity is key to Alzheimer’s disease: from ageing to dementia

**DOI:** 10.1093/brain/awaf008

**Published:** 2025-01-15

**Authors:** Léa Chauveau, Brigitte Landeau, Sophie Dautricourt, Anne-Laure Turpin, Marion Delarue, Oriane Hébert, Vincent de La Sayette, Gaël Chételat, Robin de Flores

**Affiliations:** U1237, Physiopathology and Imaging of Neurological Disorders (PhIND), Neuropresage Team, INSERM, University of Caen Normandy, GIP Cyceron, Caen 14000, France; U1237, Physiopathology and Imaging of Neurological Disorders (PhIND), Neuropresage Team, INSERM, University of Caen Normandy, GIP Cyceron, Caen 14000, France; U1237, Physiopathology and Imaging of Neurological Disorders (PhIND), Neuropresage Team, INSERM, University of Caen Normandy, GIP Cyceron, Caen 14000, France; Centre mémoire ressource recherche, Hôpital des Charpennes, Hospices civils de Lyon, Lyon 69000, France; U1237, Physiopathology and Imaging of Neurological Disorders (PhIND), Neuropresage Team, INSERM, University of Caen Normandy, GIP Cyceron, Caen 14000, France; U1237, Physiopathology and Imaging of Neurological Disorders (PhIND), Neuropresage Team, INSERM, University of Caen Normandy, GIP Cyceron, Caen 14000, France; U1237, Physiopathology and Imaging of Neurological Disorders (PhIND), Neuropresage Team, INSERM, University of Caen Normandy, GIP Cyceron, Caen 14000, France; Service de Neurologie, CHU de Caen, Caen 14000, France; U1237, Physiopathology and Imaging of Neurological Disorders (PhIND), Neuropresage Team, INSERM, University of Caen Normandy, GIP Cyceron, Caen 14000, France; U1237, Physiopathology and Imaging of Neurological Disorders (PhIND), Neuropresage Team, INSERM, University of Caen Normandy, GIP Cyceron, Caen 14000, France

**Keywords:** functional connectivity, anterior-temporal (AT)/posterior-medial (PM) systems, medial temporal lobe, Alzheimer’s disease, aging, resting-state functional MRI

## Abstract

Curing Alzheimer's disease remains hampered by an incomplete understanding of its pathophysiology and progression. Exploring dysfunction in medial temporal lobe networks, particularly the anterior-temporal (AT) and posterior-medial (PM) systems, might provide key insights, because these networks exhibit alterations in functional connectivity along the entire Alzheimer's continuum, potentially influencing disease propagation. However, the specific changes in each network and their clinical relevance across stages are not yet fully understood. This requires consideration of commonly used biomarkers, clinical progression, individual variability and age confounds.

Here, we leveraged monocentric longitudinal data from 261 participants spanning the adult lifespan and the Alzheimer's continuum. The sample included cognitively unimpaired adults aged 19–85 years (*n* = 209; 8 of 64 older adults >60 years of age were amyloid-β-positive) and amyloid-β-positive patients fulfilling diagnostic criteria for mild cognitive impairment (MCI, *n* = 26; 18 progressed to Alzheimer-dementia within 7 years) or Alzheimer's-type dementia (*n* = 26). Participants underwent structural and resting-state functional MRI, florbetapir and ^18^F-fluorodeoxyglucose-PET and global cognitive assessments, with up to three visits over a maximum period of 47 months.

Network connectivity was assessed using seed-based analyses with the perirhinal and parahippocampal cortices as seeds, within data-driven masks reflecting the AT and PM networks. Generalized additive and linear mixed models were run to assess age-specific effects and Alzheimer's-related alterations. In this context, we explored various markers of pathological and clinical severity, including cerebral amyloid uptake, glucose metabolism, hippocampal volume, global cognition, diagnostic staging and time to dementia onset.

Our findings revealed distinct patterns of connectivity linked to normal ageing or Alzheimer's disease. Advancing age throughout adulthood was associated with lower PM connectivity and more subtle changes in AT connectivity, and Alzheimer's disease was characterized by AT hyperconnectivity without global changes in PM connectivity. Specifically, AT connectivity was higher in MCI and Alzheimer-dementia patients compared with older controls and was positively associated with amyloid burden, glucose hypometabolism, hippocampal atrophy and global cognitive deficits in older adults, ranging from unimpaired to demented. Additionally, higher AT connectivity was correlated with faster progression to Alzheimer-dementia in MCI patients.

This comprehensive approach allowed us to reveal that excessive connectivity within the AT network is linked intrinsically to the pathological and clinical progression of Alzheimer's disease. These insights might guide future research to a better understanding of cascading events leading to the disease and hold promise for developing prognostic tools and therapeutic interventions targeting these specific network alterations.

## Teaser

By analysing functional connectivity within medial temporal lobe networks, Chauveau *et al.* provide evidence that hyperconnectivity within the anterior-temporal network is key to the pathophysiology and progression of Alzheimer's disease.

## Introduction

Although Alzheimer's disease is the leading cause of dementia worldwide, its biological mechanisms remain unclear, and improving its diagnosis/prognosis is crucially needed for patient care.^1^ To address these challenges, numerous studies have focused on medial temporal lobe regions, because they are the earliest cortical target of neurofibrillary tangles and neuronal loss in typical Alzheimer's disease.^2-7^ This intensive inquiry also arose from their involvement in a wide range of cognitive functions, such as declarative memory, affective processing and spatial navigation,^8-11^ that can be impaired in Alzheimer's disease.

Over the past few decades, *in vivo* neuroimaging studies have supported prior histological and animal findings arguing that disease progression is related to network organization.^2,12-15^ Brain networks are not only depicted as passive conduits through which pathology propagates; they can also act as independent drivers that actively modulate the spread of pathological proteins.^16,17^ Such network dysfunction in Alzheimer's disease might precede mesoscale structural and molecular damage, prior to symptom onset.^12,18,19^

Importantly, two functional networks have been identified at the crossroads of the medial temporal lobe: the anterior-temporal (AT) and posterior-medial (PM) systems.^8,20-22^ These networks are particularly relevant to Alzheimer's pathophysiology owing to their specific exposure to lesions, with tau pathology specifically targeting AT and amyloid plaques targeting PM.^23-27^ Despite evidence of Alzheimer-related connectivity abnormalities, the nature of these changes remains unclear owing to inconsistent study results. Although reduced functional connectivity has been described consistently for PM, findings for AT are mixed, with either increased or decreased functional connectivity across the Alzheimer's continuum.^28-37^

With the exception of one study linking these changes to CSF phosphorylated tau (p-tau) levels, white matter integrity, entorhinal cortex thickness and memory abilities in early Alzheimer's disease,^29^ comprehensive evaluations relating functional connectivity within the AT and PM networks to markers of pathological and clinical severity across the Alzheimer's continuum are lacking. In particular, assessments of common biomarkers and clinical evolution should be considered. Longitudinal data would help to predict disease progression and account for individual variations. Besides, age-specific effects have been greatly underestimated and rarely balanced against pathological processes. Studies have generally reported lower PM connectivity and either higher or unchanged AT connectivity with older age^36,38-40^; two studies presented contrasting trajectories, with PM connectivity increasing while AT connectivity decreased with age.^41,42^ Altogether, these inconsistencies make it difficult to identify a reliable Alzheimer's signature network dysfunction.

To fill these gaps, the present study provides a comprehensive overview of functional connectivity within the AT and PM networks in ageing and Alzheimer's disease, using longitudinal multimodal neuroimaging and cognitive assessments. Our aims were to investigate: (i) age-related trajectories throughout adulthood; (ii) stage differences across the Alzheimer's continuum; (iii) associations with multiple *in vivo* markers of brain lesions and cognitive impairment; (iv) relationships to dementia onset in prodromal patients; and (v) distinctions between age-specific and disease-related effects.

## Materials and methods

### Participants

The study involved 261 participants who were enrolled in the IMAP+ clinical trial between 2008 and 2016. Participants were native French speakers, right-handed, with a minimum of 7 years of education, and had no history of alcoholism, drug abuse, head trauma, neurological or psychiatric disorder. The IMAP+ trial received approval from the regional ethics committee (Comité de Protection des Personnes Nord-Ouest III, Caen) and is registered with ClinicalTrials.gov (number: NCT01638949). All participants gave written informed consent to the study before the investigation.

Our sample included 209 cognitively unimpaired adults aged 19–85 years and 52 patients on the Alzheimer's continuum. Cognitively unimpaired adults were recruited from the community using flyers and advertisements in local newspapers. Their cognitive performances were within the normal range for their age and education level, based on a battery of standardized tests assessing global cognition, depression, language, episodic memory, executive functioning and visuospatial and motor skills (see Supplementary material, ‘Methods’ section). No participants expressed memory complaints. Patients were recruited from local memory clinics and selected according to internationally agreed criteria. Amnesic patients with mild cognitive impairment (MCI) satisfied Petersen's criteria (*n* = 26).^43^ Patients with Alzheimer-dementia fulfilled the standard National Institute of Neurological and Communicative Disorders and Stroke and the Alzheimer's Disease and Related Disorders Association (NINCDS-ADRDA) clinical criteria for mild to moderate probable Alzheimer's disease (*n* = 26).^44^ Clinical diagnosis was assigned by consensus under the supervision of a senior neurologist (V.d.l.S.).

Participants were followed for ≤4 years, with one to three assessments of neuroimaging and cognitive data (±18 months apart). Participants >60 years of age underwent an ^18^F-florbetapir PET examination to assess amyloid-β (Aβ) deposition. Among cognitively unimpaired older adults, the prevalence of Aβ-positive was 12.5% (8 of 64). Patients across the Alzheimer's continuum were all confirmed to be Aβ-positive (see section ‘Definition of amyloid-β-status’). Sample characteristics are reported in Table 1.

**Table 1:** Cohort demographics

Characteristic	Unimpaired young and middle-aged adults	Unimpaired older adults	MCI patients	AD-demented patients
*n*	145	64	26	26
Age at baseline (years)	36.9 ± 12.2 [19.5, 59.6]	69.8 ± 6.56 [60, 84.6]	74.5 ± 6.7 [60.7, 85]	67.2 ± 9.8 [51.7, 84.1]
Sex (F/M)	75/70	36/28	8/18	11/15
Years of education	13.9 ± 2.9 [9, 20]	12.1 ± 3.7 [7, 20]^a^	11.8 ± 4.3 [6, 20]	11.5 ± 3.6 [7, 20]
Aβ (+/−)	–	8/56	26/0	26/0
MMSE at baseline	29.4 ± 0.7 [27, 30]	28.8 ± 1.2 [26, 30]	26.6 ± 1.7 [22, 30]	20.6 ± 4.9 [12, 29]
MDRS at baseline	142.2 ± 2.4 [132, 144]	141.2 ± 2.7 [130, 144]	132.3 ± 5.6 [122, 142]	118.2 ± 10.8 [101, 139]
Baseline to follow-up fMRI (months)	15.4 ± 8.6 [0, 47.2]	25.1 ± 13.2 [0, 41.2]	17.9 ± 13.4 [0, 40.7]	11.6 ± 9.4 [0, 21.6]
Number of available time points	1.8 ± 0.4 [1, 2]	2.3 ± 0.7 [1, 3]	1.9 ± 0.7 [1, 3]	1.6 ± 0.5 [1, 2]
fMRI to ^18^F-fluorodeoxyglucose-PET (months)	–	0.9 ± 1.3 [0, 8]	0.5 ± 0.7 [0, 3.6]	0.8 ± 1.1 [0, 5.8]
fMRI to AV45-PET (months)	–	2.1 ± 4.8 [0, 45.8]	2.1 ± 2.8 [0.1, 9.4]	1.1 ± 2.1 [0, 10.5]
Converters to AD-dementia	–	–	18	–
Clinical follow-up (months)	–	–	31.5 ± 25.5 [0, 85.4]	–

Continuous variables are indicated as follows: mean ± standard deviation [minimum, maximum]. Aβ = amyloid-β; AD = Alzheimer's disease; F = female; M = male; fMRI = functional MRI; MCI = mild cognitive impairment; MDRS = Mattis Dementia Rating Scale; MMSE = Mini-Mental State Examination.

^a^Education level was missing for one participant.

Patients included as MCI were followed clinically until December 2022. From this sample, 69% (18 of 26) progressed from MCI to Alzheimer-dementia within 7 years. Dementia diagnosis was posited in a staff meeting by a senior neurologist (V.d.l.S.) according to standard clinical criteria and based on feedback from the patient’s clinicians. Clinical data came from medical reports and possible neuropsychological evaluations over the last few years, and included information on autonomy, daily life, cognition and general health.

### Neuroimaging acquisition

Participants were scanned repeatedly with the same MRI and PET scanners at the Cyceron Center (Caen, France): a Philips Achieva 3.0 T scanner and a Discovery RX VCT 64 PET-CT device (General Electric Healthcare), respectively.

#### MRI acquisition

T1-weighted anatomical images were acquired using a 3D fast-field echo sequence (3D-T1-FFE sagittal; SENSE factor = 2; repetition time = 20 ms; echo time = 4.6 ms; flip angle = 10°; 180 slices with no gap; slice thickness = 1 mm; field of view = 256 mm × 256 mm; in-plane resolution = 1 mm × 1 mm).

Resting-state functional MRI (fMRI) scans were obtained using an interleaved two-dimensional T2* SENSE EPI sequence designed to reduce geometric distortions (2D-T2*-FFE-EPI axial, SENSE = 2; repetition time = 2382 ms; echo time = 30 ms; flip angle = 80°; 42 slices with no gap; slice thickness = 2.8 mm; field of view = 224 mm × 224 mm; in plane resolution = 2.8 mm × 2.8 mm; 280 volumes, acquisition time = 11.5 min). Of note, 11 scans from cognitively unimpaired adults <60 years of age were acquired using a slightly different sequence (2D-T2*-FFE-EPI axial, SENSE = 2; repetition time = 2200 ms; echo time = 35 ms; flip angle = 80°; 35 slices with no gap; slice thickness = 3.5 mm; field of view = 224 mm × 224 mm; in plane resolution = 3.5 mm × 3.5 mm; 300 volumes, acquisition time = 11.11 min). The difference between sequences was controlled in the relevant statistical analyses. During acquisition, participants wore earplugs, and their heads were stabilized with foam pads to minimize head motion. The light in the scanner room was turned off, and participants were asked to relax by lying down and closing their eyes, without falling asleep. A short debriefing after the acquisition ensured that participants had no difficulty staying awake and that nothing distracted them.

Non-echo planar imaging (EPI) T2* volumes (2D-T2*-FFE axial; SENSE factor = 2; repetition time = 3514 ms; echo time = 30 ms; flip angle = 90°; 70 slices with no gap; slice thickness = 2 mm; field of view = 256 mm × 256 mm; in-plane resolution = 2 mm × 2 mm) were additionally obtained for fMRI pre-processing steps.

#### PET acquisition

Both ^18^F-fluorodeoxyglucose and florbetapir PET scans were acquired (resolution = 3.76 × 3.76 mm × 4.9 mm; field of view = 157 mm; voxel size = 1.95 mm × 1.95 mm × 3.27 mm). A transmission scan was performed for attenuation correction before the PET acquisition.

For ^18^F-fluorodeoxyglucose-PET, participants fasted for ≥6 h before scanning and remained in a quiet, dark environment for 30 min prior to the radiotracer injection. The PET acquisition, which lasted 10 min, started 50 min after the intravenous injection of 5.4 mCi of ^18^F-fluorodeoxyglucose.

For florbetapir-PET, data were reconstructed locally into 4 × 5 min frames for the 50–70 min interval following the injection of 4 MBq/kg of florbetapir.

### Neuroimaging preprocessing

#### Functional MRI preprocessing

EPI data were initially checked visually for movement, lesions, abnormalities, head misplacement and signal loss (especially given that temporal regions can be particularly affected). The TSDiffana routine (http://imaging.mrc-cbu.cam.ac.uk/imaging/DataDiagnostics) was applied to functional raw volume to identify artefacts. Data showing evidence for significant head motion, defined as translation >3 mm (maximum motion) or rotation >1.5° (mean absolute Euler angle), were excluded.

Preprocessing steps included slice timing correction, realignment to the first volume, rigid coregistration onto T1 images, and spatial normalization using SPM12. Individual T1-to-EPI coregistration was performed as followed: (i) EPI onto non-EPI T2*; (ii) non-EPI T2* onto T2; and (iii) T2 onto T1. The EPI image was warped to align with the non-EPI T2* volume to reduce geometrical distortion.^45^ Warping parameters from EPI-to-non-EPI were then combined with normalization parameters obtained from previously segmented T1 images onto the Montreal Neurological Institute (MNI) template and applied to the coregistered T1, non-EPI T2* and EPI volumes.

Resulting images (distortion corrected in MNI space) were smoothed with a 4 mm full width at half maximum Gaussian kernel, then bandpass filtered (0.01–0.08 Hz) using the Analysis of Functional NeuroImages (AFNI) program.

#### PET preprocessing

PET data were initially coregistered onto their corresponding T1-weighted MRI, then normalized to the MNI space using the parameters from the T1 segmentation procedure. Resulting images were quantitatively normalized using either cerebellum or white matter uptake as reference, depending on analyses (cross-sectional versus longitudinal, respectively).

### Definition of amyloid-β status

The cerebral Aβ-positivity threshold was calculated as the 99.9th percentile of the standardized uptake value ratio (SUVR) distribution from 45 participants younger than 40 years, corresponding to an SUVR of 1.31. Aβ status was determined primarily from the baseline scan for most of our population. However, in cases where no PET scan was available at baseline and the SUVR from subsequent scans fell below the threshold, individuals were considered Aβ-negative at baseline (*n* = 21). Given that determination of status occurred at a single time point, global neocortical amyloid^46^ levels were extracted from cerebellum-scaled PET images.

### Delineation of medial temporal lobe regions

The hippocampus, perirhinal and parahippocampal cortices were segmented automatically on T1-weighted scans using the Automatic Segmentation of Hippocampal Subfields (ASHS)^47^ software (atlas: ashsT1_atlas_upennpmc_07202018) to account for dura mater confounding and inter-individual anatomical variability in the medial temporal lobe.^48^ The standard ASHS-T1 pipeline was applied in participants with a single T1, whereas the Longitudinal Automatic Segmentation of Hippocampal Subfields (LASHiS)^49^ pipeline was used for participants with multiple scans. Each segmentation was inspected visually. Failed segmentations were edited manually if they involved lateral hippocampal segmenting errors in coronal slices (∼30%) or excluded. Left and right structures were merged to limit the number of analyses.

### Functional connectivity assessment

#### Seed-based analyses

**Figure 1:**
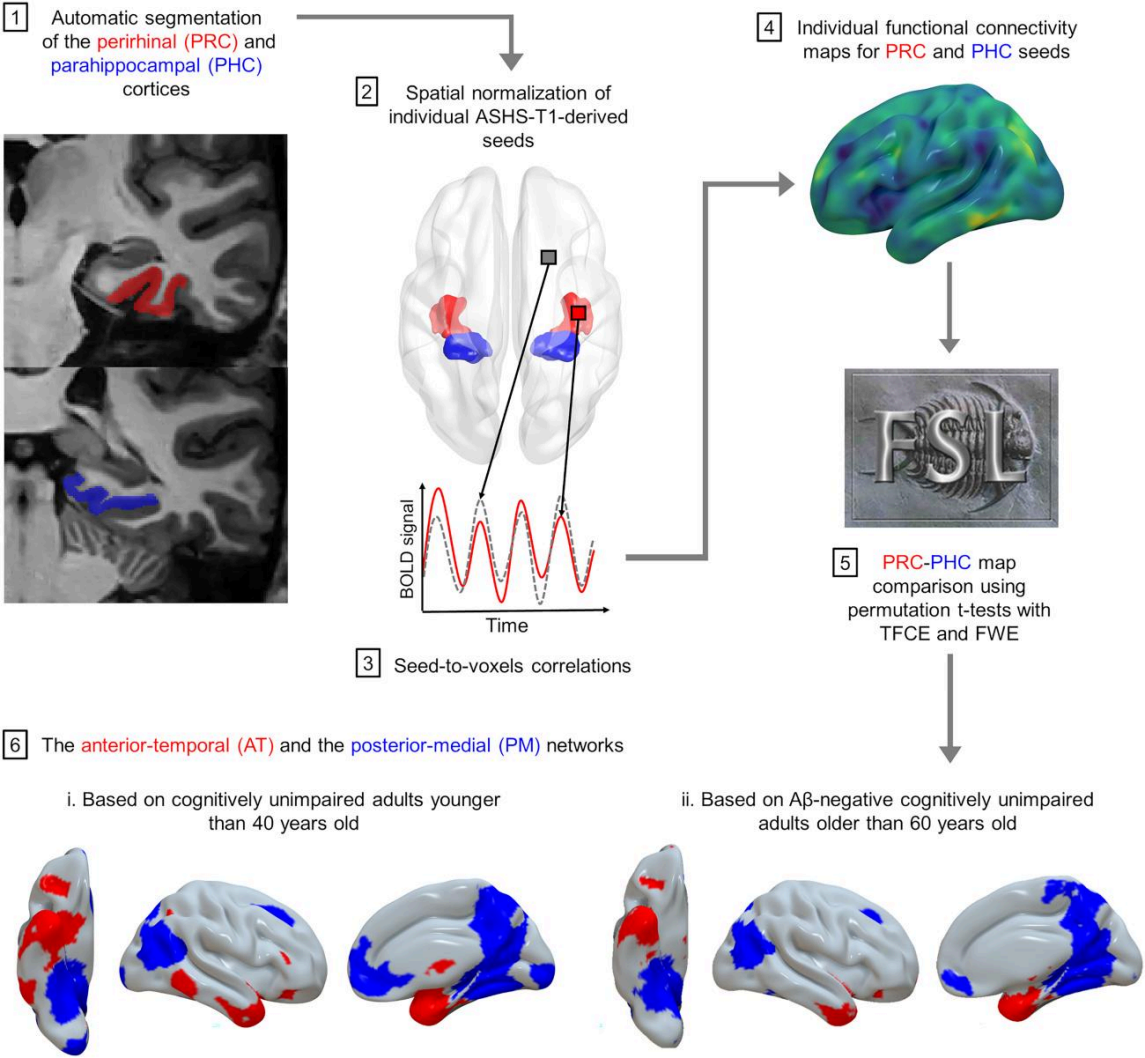
**The anterior-temporal and posterior-medial networks.** Summary of the identification procedure steps. Aβ = amyloid-β; ASHS = Automatic Segmentation of Hippocampal Subfields software; BOLD = blood oxygen level-dependent; FWE = family-wise error; TFCE = threshold-free cluster enhancement.

Resting-state fMRI scans were processed using SPM12 and the MarsBaR toolbox (version 0.44). The AT and PM networks were defined following the procedure described in Fig. 1. The perirhinal (consisting of Brodmann areas 35 and 36) and parahippocampal cortices were normalized to MNI space and used as seeds, because they are the hub regions of the AT and PM networks and best suited for accurate discrimination.^8^ Positive correlations between the mean time course in individual ASHS-T1-derived seeds and the time course in each grey matter voxel were assessed, resulting in two connectivity maps per scan: one for the perirhinal cortex and one for the parahippocampal cortex. To account for signal loss in resting-state fMRI data, we excluded voxels with substantial dropout by creating a grey matter mask derived from the T1 and non-EPI T2* segmentations, thresholded at 0.25, then combined. Low-frequency drifts were removed for each seed, and the mean time courses in white matter, CSF, whole brain, their derivatives, and six head motion parameters from realignment were regressed out to remove potential sources of spurious variance. A Fisher *z*-transformation and a 6.3 mm full width at half maximum Gaussian smoothing were finally applied to connectivity maps.

#### Mask creation and connectivity extraction

The following mask procedure was carried out exclusively on healthy populations relevant to each analysis. More precisely, masks intended for age-related analyses were obtained from baseline data of cognitively unimpaired adults <40 years of age, whereas masks intended for Alzheimer-related analyses were obtained from baseline data of Aβ-negative cognitively unimpaired adults >60 years of age (see Fig. 1).

To identify regions specific to the AT and PM networks, we compared perirhinal and parahippocampal functional connectivity maps using permutation *t*-tests. One-sample permutation *t*-tests were initially conducted separately on the perirhinal and parahippocampal maps, using the FSL randomize command with threshold-free cluster enhancement (TFCE) and family-wise error (FWE) correction.^50^ This step allowed us to retain only regions significantly connected to each seed (*P*_FWE_corr_ < 0.05). Then, paired permutation *t*-tests were performed to compare perirhinal-to-parahippocampal (and vice versa) connectivity maps, using the same settings (*P*_FWE_corr_ < 0.05, TFCE). This step aimed to highlight regions specific to AT (more strongly connected to the perirhinal seed) and PM (more strongly connected to the parahippocampal seed). Lastly, the AT mask was created by combining the outputs of the perirhinal one-sample and perirhinal-to-parahippocampal paired *t*-tests, while the PM mask consisted of the overlap between the parahippocampal one-sample and parahippocampal-to-perirhinal paired *t*-test results.

Individual grey matter masks were generated for each participant based on their respective T1 grey matter segmentation, thresholded at 0.5. Group-level AT and PM masks were multiplied by these individual grey matter masks to create participant-specific masks tailored to the AT and PM networks. This approach accounted for inter-individual anatomical variability to enhance accuracy and minimize signal noise arising from voxels in CSF or white matter. Seeds were removed from the masks to prevent autocorrelations.

Mean connectivity was extracted from unsmoothed perirhinal and parahippocampal connectivity maps within participant-specific AT and PM masks, respectively, for all available data. The average signal was computed using positive voxels only, because negative values were minimal and probably represented non-biologically meaningful noise.^51^ These metrics served as proxies for the global functional connectivity of the AT or PM networks, specifically reflecting perirhinal-to-AT and parahippocampal-to-PM connectivity.

##### Network parcellation

To examine regional differences in connectivity, AT and PM masks were split according to the Brainnetome atlas parcellation.^52^ This resulted in 43 regions of interest (ROIs) for AT and 49 for PM for the young adult-derived masks (Supplementary Tables 1 and 2) and 24 ROIs for AT and 41 for PM for the older adult-derived masks (Supplementary Tables 3 and 4). Only ROIs including ≥100 voxels were investigated. The average connectivity within each ROI was then extracted from unsmoothed connectivity maps, reflecting seed-to-ROI specific connectivity.

### Calculation of amyloid uptake, glucose metabolism, hippocampal volume and global cognition

Amyloid uptake and glucose metabolism were quantified by extracting SUVRs from white matter-scaled PET images in all available data. These extractions were conducted within individual-specific grey matter masks in Alzheimer-sensitive regions, previously identified as exhibiting high amyloid burden or severe hypometabolism in patients with Alzheimer's disease.^46^ Hippocampal volumes were computed by combining segmentation outputs for the anterior and posterior hippocampus. Subsequently, volumes were normalized by the total intracranial volume (TIV) to correct for differences in head size among participants [normalized volume = raw volume/(TIV × 1000)]. Global cognition was assessed using the Mini-Mental State Examination (MMSE) and Mattis Dementia Rating Scale (MDRS) at each visit.

### Statistical analyses

Statistical analyses were conducted in R version 4.2.0, using the following libraries: lmer4, gamm4, mgcv and emmeans. Figures were computed using the ggplot2 R package or the BrainNet Viewer software version 1.6.

Unless otherwise stated, all analyses were conducted on longitudinal data using linear mixed models owing to their ability to handle within-subject observations and missing data and to address unbalanced designs. For analyses of trajectories, generalized additive (mixed) models were initially run to explore non-linear relationships without *a priori* inferences. Mixed models were fitted to the data using restricted maximum likelihood estimation and included by-participant intercepts. Adjustments were made for sex, years of education and age. The significance of fixed effects was assessed using Type II or III *F*-tests, with degrees of freedom approximated using Satterthwaite's method. In cases involving multiple comparisons, results were deemed significant following the Holm–Bonferroni controlling procedure^53^ (*P*_HB_corr_ < 0.05).

Age effects on functional connectivity were assessed among cognitively unimpaired adults (aged 19–85 years), excluding Aβ-positive older adults. These analyses were performed using longitudinal data and the set of masks defined in young adults. Separate mixed models were run for AT and PM. Average connectivity within each network served as the dependent variable, and age (calculated as the time difference between birth date and fMRI examination) served as a predictor. Difference in fMRI sequence parameters was incorporated as an additional covariate. Similar models were also conducted at the ROI level to outline regional differences within the networks.

Differences in functional connectivity between Alzheimer's stages were investigated in participants >60 years of age, with and without cognitive impairment, using the masks defined in older adults. Baseline differences among groups, categorized by clinical diagnosis and Aβ status, were compared using ANCOVA owing to the cross-sectional nature of these analyses. Pairwise comparisons were conducted as *post hoc* analyses using independent *t*-tests based on estimated marginal means. Longitudinal changes over time were assessed in mixed models that incorporated the interaction between groups and the time between baseline and follow-up fMRI examinations as an additional fixed effect.

Associations with neuroimaging and cognitive features of Alzheimer's disease were examined using time-adjusted models. Separate mixed models were constructed for each network and feature of interest (amyloid uptake, glucose metabolism, hippocampal volume, MMSE and MDRS scores), resulting in 10 tests. The same associations were also tested at the ROI level to examine regional differences within the networks. Functional connectivity was also tested in relationship to time from prodromal to dementia stage in MCI converters. Note that although the analyses were conducted using longitudinal data, the presented results do not account for time interactions, potentially giving the impression of cross-sectional findings. Our primary focus was on identifying how neuroimaging and cognitive features of Alzheimer's disease influence AT/PM functional connectivity. Using longitudinal data accounts for individual variability, enhancing the precision of our estimates and revealing more reliable associations between neuroimaging and cognitive features of Alzheimer's disease and network connectivity.

Incremental effects of age and Alzheimer's disease on functional connectivity were investigated using a hierarchical approach, by ranking groups according to prior evidence of the Alzheimer's continuum.^54-56^ Generalized additive models were applied to baseline data, with group included as a smooth term (using *k* = 6 basis dimensions to control the flexibility of the smooth term) to derive connectivity trajectories across the whole sample. Group differences in longitudinal changes over time were assessed by including an interaction term between time and group in the fixed effects of linear mixed models. Analyses were repeated using values extracted from both sets of masks (see ‘Results’ section and Supplementary Table 5).

All analyses were replicated using models that integrated both networks simultaneously to explore interactions between AT and PM and to strengthen our findings. These models included connectivity values as the dependent variable and the interaction between networks and the variable of interest as predictors, along with the covariates relevant to each analysis (Supplementary Table 6). Lastly, we repeated all analyses by adjusting for mean framewise displacement (FD) to ensure that the observed effects were not influenced by residual motion differences (see Supplementary material). Note that the summary of framewise displacement among study groups can be found in Supplementary Table 7.

## Results

### Description of the anterior-temporal and posterior-medial networks

The networks identified in both sets of masks (Fig. 1) aligned with previous literature.^8,20-23,57^ They intersected the hippocampus along its longitudinal axis and included regions such as the lateral entorhinal cortex, amygdala, temporopolar and orbitofrontal cortices for AT, and the medial entorhinal cortex, retrosplenial, posterior cingulate, precuneus, ventromedial prefrontal cortex, and angular, fusiform and inferior gyri for PM (based on visual inspection cross-referenced with the *Atlas of the Human Brain*^58^).

Comparison of masks derived from younger adults and older adults revealed that the younger adult masks were more expansive [32 566 versus 23 343 voxels included for AT (Dice coefficient = 0.71) and 67 120 versus 63 167 for PM (Dice coefficient = 0.78)], suggesting a loss of spatially connected regions with age. Specifically, the younger adult masks included additional regions in the parietal lobules, frontal gyri and basal ganglia for AT, and in the occipital lobe, superior frontal gyrus and postcentral gyrus for PM.

### Age-related trajectories

**Figure 2:**
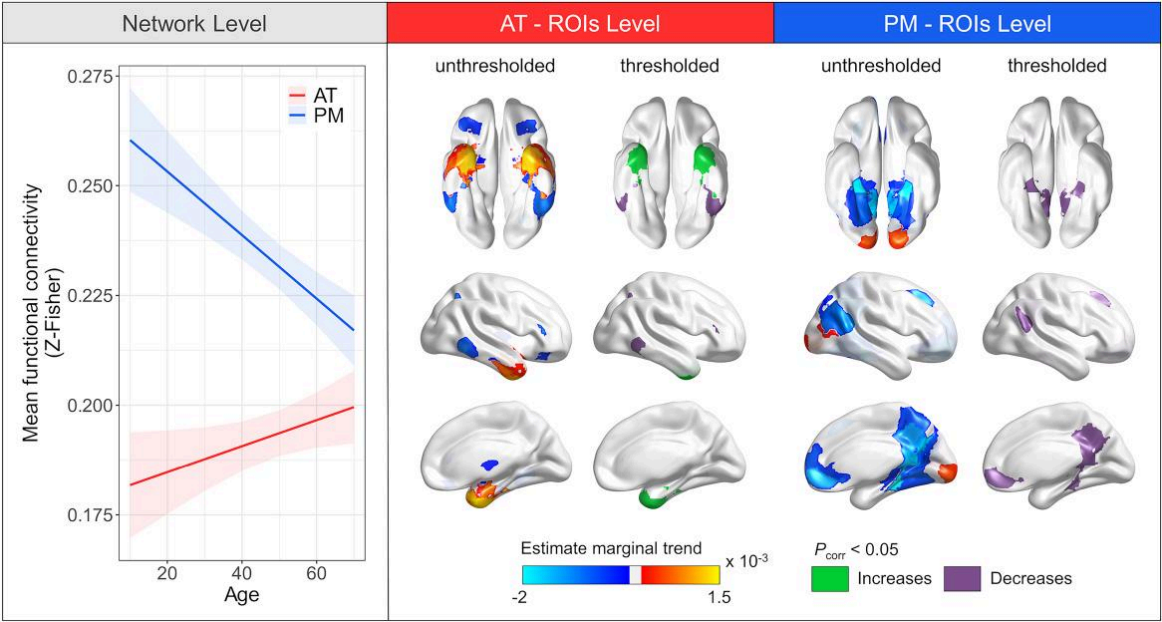
**Age-related trajectories of functional connectivity in the anterior-temporal and posterior-medial networks across the adult lifespan.** The network-level panel (*left*) represents age-specific effects on anterior-temporal (AT) and posterior-medial (PM) global functional connectivity. Connectivity of both networks was related to age (*P* < 0.05, no framewise displacement adjustment). Regression lines indicate model-derived estimates. Shaded areas represent the 95% confidence interval. Raw data-points are presented in Supplementary Fig. 1. The regions of interest (ROIs)-level panel (*right*) represents age-specific effects on mean seed connectivity within AT and PM ROIs. Unthresholded maps reflect model-derived estimates for all corresponding analyses, including non-significant ones. Thresholded maps represent ROIs for which the *P*-value associated with the age term survived to Holm–Bonferroni adjustment for multiple testing (*P*_HB_corr_ < 0.05). Green indicates positive associations between age and functional connectivity, and purple indicates negative associations.

Age trajectories for AT and PM functional connectivity across the adult lifespan were examined initally using generalized additive mixed models, which provided no support for non-linearity (effective degrees of freedom = 1). Linear mixed models (*n* = 200, data = 389) also indicated that AT functional connectivity was higher [*F* = 4, *P* = 0.046, *β* = 0.0003, 95% confidence interval (CI) = 0.0000, 0.0006], whereas PM functional connectivity was lower (*F* = 25.3, *P* < 0.001, *β* = −0.0007, 95% CI = −0.0010, −0.0004; Fig. 2) with age. After adjusting for FD, only PM connectivity remained associated with age (Supplementary Table 8). Likewise, in the model including both networks simultaneously, *post hoc* analyses indicated an age effect solely on PM connectivity (Supplementary Table 6).

Additional analyses were conducted to investigate the effects of age more locally. Within AT, perirhinal connectivity was higher with advancing age in anterior temporal regions, such as the anterior hippocampus, lateral entorhinal cortex, planum temporal, temporal pole, fusiform, and inferior and middle temporal gyri. In contrast, it was lower in posterior regions of the fusiform and temporal gyri and extra-temporal regions, including parts of the superior parietal lobule, and postcentral and inferior frontal gyri. Within PM, parahippocampal connectivity was negatively associated with age across the whole network (Fig. 2 and Supplementary Tables 9 and 10).

### Differences among Alzheimer's stages

**Figure 3:**
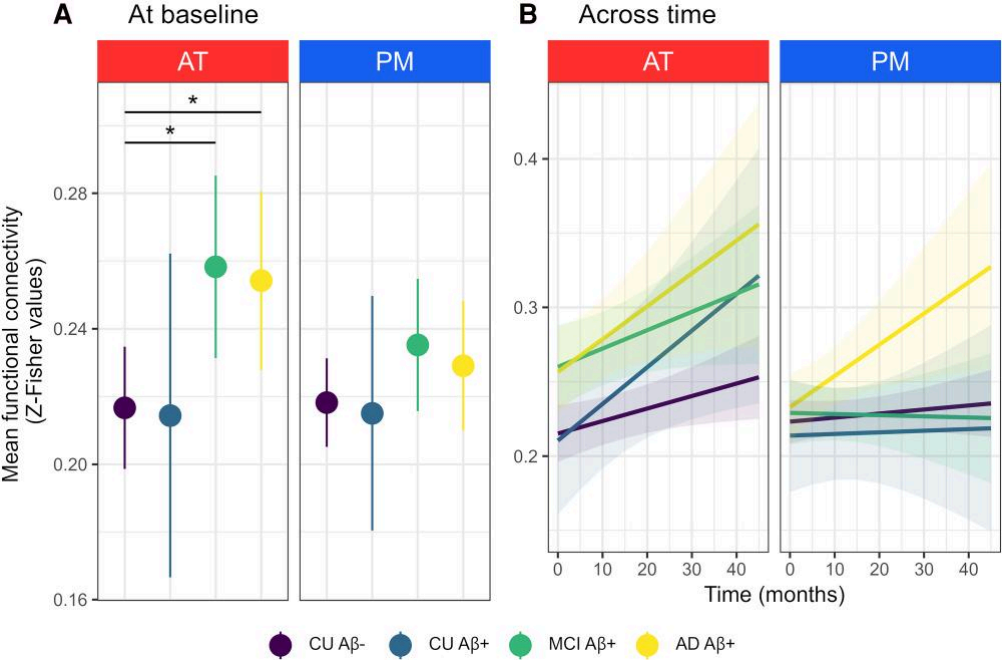
**Group differences in functional connectivity within the anterior-temporal and posterior-medial networks in older adults, from healthy to Alzheimer-demented.** (**A**) Only anterior-temporal (AT) connectivity differed by group at baseline (*P* < 0.05, no framewise displacement adjustment). Point ranges indicate model-derived estimates with 95% confidence intervals. *P < 0.05 as results of *post hoc t*-tests. Raw data-points are presented in Supplementary Fig. 2. (**B**) No significant Time × Group interaction was found for AT or posterior-medial (PM) using longitudinal data. Regression lines indicate model-derived estimates trend. Shaded areas represent 95% confidence intervals. Raw data-points are presented in Supplementary Fig. 3. Aβ = amyloid-β; AD = Alzheimer's disease; CU = cognitively unimpaired; MCI = mild cognitive impairment.

Baseline analyses (*n* = 115) revealed between-group differences for AT (*F* = 3.3, *P =* 0.02), but not for PM (*F* = 0.9, *P* = 0.45). *Post hoc* comparisons indicated that Aβ-positive patients with MCI or Alzheimer-dementia exhibited greater functional connectivity in AT compared with Aβ-negative controls (estimate ± SE = 0.0416 ± 0.0168, *t* = 2.5, *P* = 0.01; estimate ± SE = 0.0375 ± 0.0161, *t* = 2.3, *P* = 0.02, respectively). We found no significant differences between Aβ-positive and Aβ-negative cognitively unimpaired older adults or between Aβ-positive patients with MCI and Alzheimer-dementia (Fig. 3A).

Longitudinal analyses (*n* = 115, data = 240) revealed no significant interactions between time and group for either AT (*F* = 1, *P* = 0.4) or PM (*F* = 1.5, *P* = 0.2) (Fig. 3B).

To prevent any bias from the small sample size of Aβ-positive cognitively unimpaired older adults affecting model power, we conducted additional analyses by: (i) merging Aβ-positive and Aβ-negative cognitively unimpaired older adults; and (ii) excluding Aβ-positive cognitively unimpaired older adults. Results regarding baseline differences between older individuals with or without cognitive impairment remained consistent across approaches (see Supplementary Tables 11 and 12). Only the effects from models merging Aβ-positive and Aβ-negative cognitively unimpaired individuals survived FD correction (Supplementary Table 1^1^).

### Associations with Alzheimer's disease neuroimaging biomarkers and global cognition

**Figure 4:**
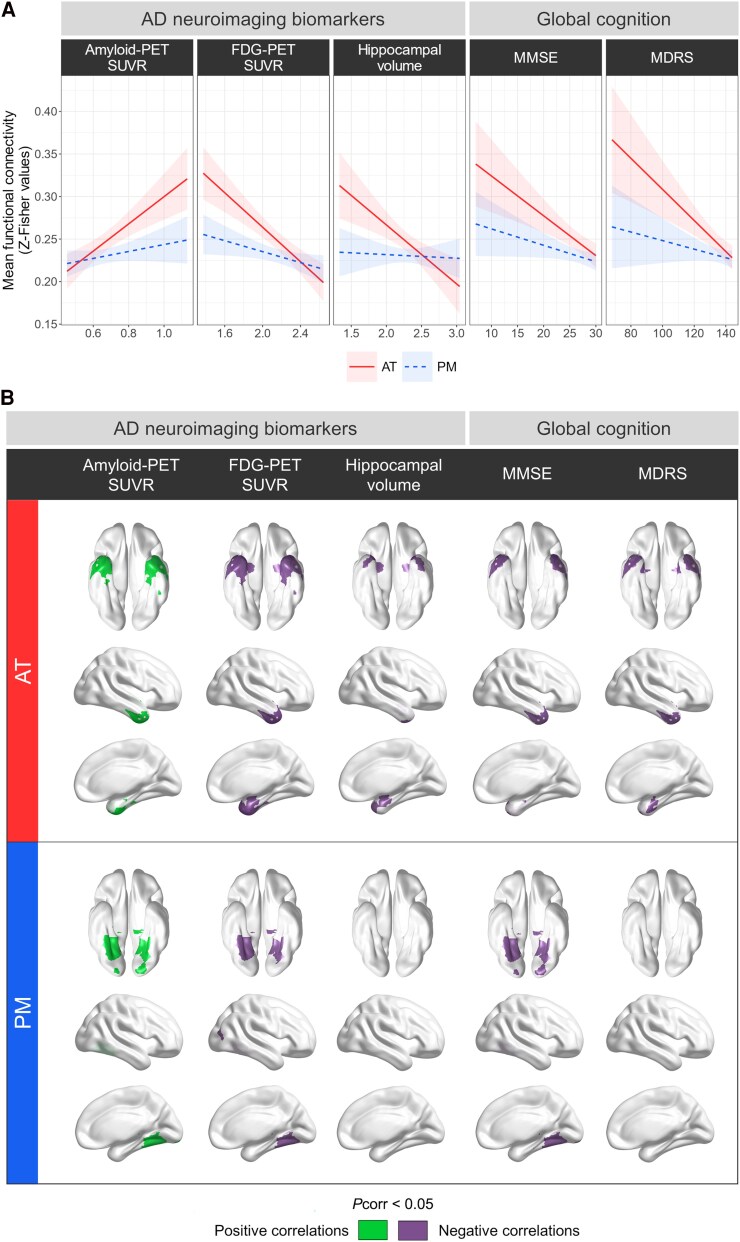
**Associations between functional connectivity within the anterior-temporal and posterior-medial networks and neuroimaging or cognitive markers of Alzheimer's disease.** (**A**) Relationships between anterior-temporal (AT) and posterior-medial (PM) global connectivity and Alzheimer’s-associated biomarkers and clinical scores. Filled lines represent significant associations and dashed lines non-significant ones (*P*_corr_ > 0.05, no framewise displacement adjustment). Raw data-points are presented in Supplementary Figs 4–8. (**B**) Brain maps highlighting Brainnetome-based regions of interest (ROIs) for which the *P*-value associated with the term of interest survived to Holm–Bonferroni correction (*P*_corr_ > 0.05). Green indicates positive correlations between the feature of interest and functional connectivity, and purple indicates negative correlations. AD = Alzheimer's disease; FDG = ^18^F-fluorodeoxyglucose; MDRS = Mattis Dementia Rating Scale; MMSE = Mini-Mental State Examination; SUVR = standardized uptake value ratio.

Results showed that AT (all *P*_corr_ < 0.01) but not PM (all *P*_corr_ > 0.1) connectivity was associated with all available features of Alzheimer's disease (Fig. 4A). Specifically, higher functional connectivity in AT was associated with higher amyloid uptake (*F* = 17.3, *P*_corr_ < 0.001, *β* = 0.1601, 95% CI = 0.0858, 0.2344, *n* = 114, data = 206) and lower glucose metabolism (*F* = 28.2, *P*_corr_ < 0.001, *β* = −0.1013, 95% CI = −0.1381, −0.0645, *n* = 112, data = 218) in Alzheimer-sensitive regions, smaller hippocampal volume (*F* = 12.0, *P*_corr_ = 0.005, *β* = −0.0694, 95% CI = −0.1083, −0.0306, *n* = 114, data = 237) and worse MMSE (*F* = 13.4, *P*_corr_ = 0.002, *β* = −0.0047, 95% CI = −0.0072, −0.0022, *n* = 114, data = 237) and MDRS (*F* = 15.2, *P*_corr_ = 0.001, *β* = −0.0018, 95% CI = −0.0027, −0.0009, *n* = 113, data = 224) scores. All effects remained significant after FD adjustment (Supplementary Table 8) and when testing both networks within the same models (Supplementary Table 6).

At a more local level, ROI-based analyses revealed that most of the AT regions exhibited associations with all features of interest: amyloid uptake (number of ROIs involved = 11, *F* = 8.8–24.6, *P*_corr_ < 0.05 to 0.001), glucose metabolism (*n* = 18, *F* = 8.5–31.4, *P*_corr_ < 0.05 to 0.001), hippocampal volume (*n* = 8, *F* = 9.6–21.9, *P*_corr_ < 0.05 to 0.001), MMSE (*n* = 7, *F* = 11–28.1, *P*_corr_ < 0.05 to 0.001) and MDRS (*n* = 9, *F* = 9.2–27.8, *P*_corr_ < 0.05 to 0.001) scores (Fig. 4B; Supplementary Tables 13–17). The regions were more precisely located in the anterior hippocampus, lateral entorhinal cortex, temporal pole, planum temporal, fusiform, and inferior, middle and superior temporal gyri according to the *Atlas of the Human Brain*.^58^ Of interest, higher amyloid uptake (*n* = 6, *F* = 11–20, *P*_corr_ < 0.05 to 0.001), lower glucose metabolism (*n* = 5, *F* = 11.4–17.3, *P*_corr_ < 0.05 to 0.001) and lower MMSE scores (*n* = 6, *F* = 11.3–18.9, *P*_corr_ < 0.05 to 0.001) were also correlated with higher connectivity between the parahippocampal cortex and several temporal regions within PM (Fig. 4B; Supplementary Tables 18–22). These regions were part of the medial entorhinal cortex, pulvinar thalami, and medial occipito-temporal, inferior temporal and fusiform gyri.^58^

### Association with delay to Alzheimer-dementia onset

**Figure 5:**
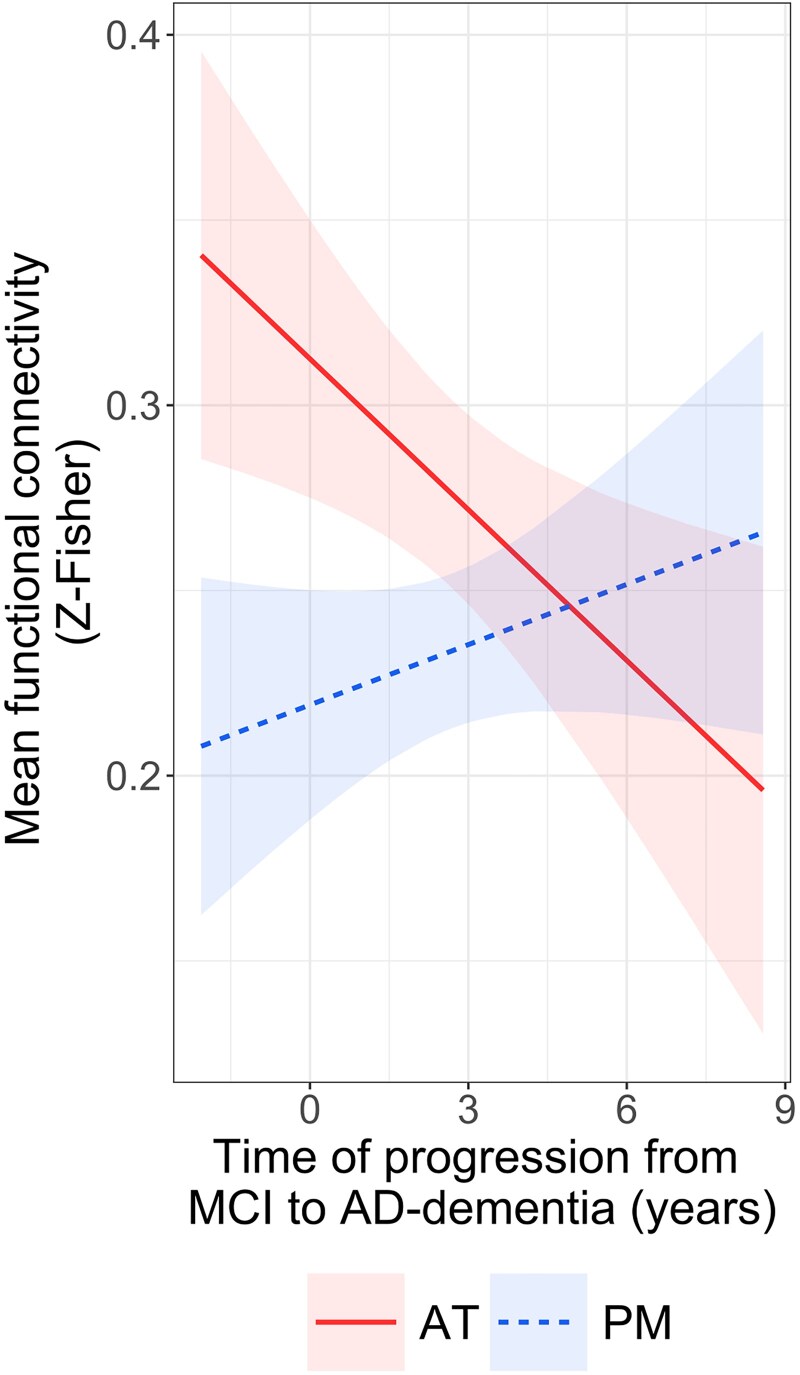
**Associations between functional connectivity in the anterior-temporal and posterior-medial networks and delay to Alzheimer-dementia onset in patients with mild cognitive impairment.** Regression lines indicate model-derived estimates. Shaded areas represent a 95% confidence interval. The continuous line represents a significant association and the dashed line a non-significant one (*P* > 0.05, no framewise displacement adjustment). Raw data-points are presented in Supplementary Fig. 9. AD = Alzheimer's disease; AT = anterior-temporal; MCI = mild cognitive impairment; PM = posterior-medial.

Within ≤7 years of clinical follow-up, 69% of Aβ-positive MCI patients evolved to Alzheimer-dementia. Investigation of the time between fMRI examination and dementia onset in relationship to AT and PM functional connectivity (*n* = 18, data = 76) revealed that higher AT functional connectivity was associated with faster progression from MCI to Alzheimer-dementia (*F* = 6.7, *P* = 0.02, *β* = −0.0136, 95% CI = −0.0229, −0.0046), whereas no significant association was found for PM (*F* = 1.6, *P* = 0.2; Fig. 5). Results were consistent when testing both networks simultaneously (Supplementary Table 6) and exhibited the same trend after adjusting for FD (Supplementary Table 8).

### Differential vulnerabilities to age and Alzheimer's disease

**Figure 6:**
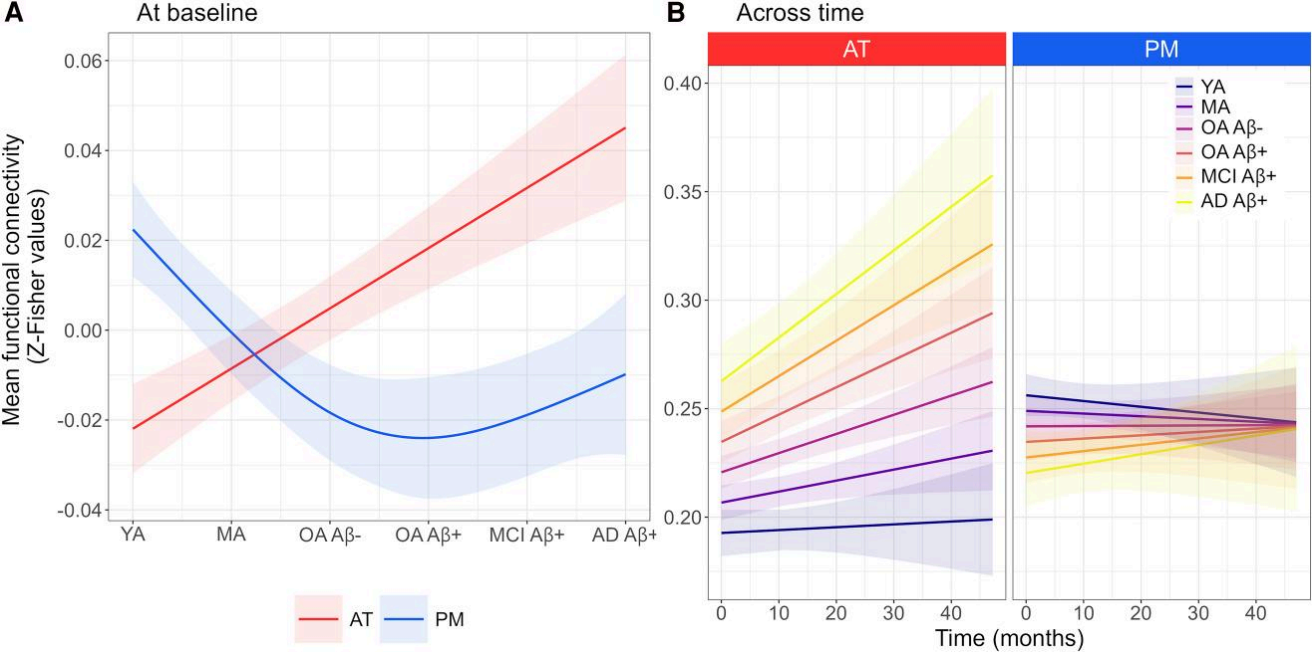
**Functional connectivity in the anterior-temporal and posterior-medial networks across the entire sample.** (**A**) At baseline, visual inspection of smooth terms indicated a significant relationship between connectivity of both networks and group ranking (*P* < 0.001, no framewise displacement adjustment). Regression lines represent partial effects determined by model-derived estimates. (**B**) Over time, there was a significant increase in functional connectivity within anterior-temporal (AT) across the entire sample range, whereas no significant change was observed in posterior-medial (PM) (*P* < 0.05, no framewise displacement adjustment). Continuous lines indicate model-derived estimates. Shaded areas represent a 95% confidence interval. Aβ = amyloid-β; AD = Alzheimer's disease; MA = middle-aged adults (40–60 years); MCI = mild cognitive impairment; OA = older adults (>60 years); YA = young adults (19–39 years).

When assessing connectivity trajectories across the whole sample, cross-sectional analyses (*n* = 259) demonstrated higher AT connectivity from young cognitively unimpaired to older demented adults (*F* = 36.0, *P* < 0.001, effective degrees of freedom = 1). In contrast, PM connectivity was lower during normal ageing but slightly higher across the Alzheimer's continuum (*F* = 10.1, *P* < 0.001, effective degrees of freedom = 2.3; Fig. 6A). Trajectories remained consistent across the masks for both younger and older adults (Supplementary Table 5).

Longitudinal analyses (*n* = 260, data = 498) revealed a significant interaction between ranked groups and time for AT (*F* = 7.3, *P=* 0.007), suggesting that AT functional connectivity increased more rapidly over time when progressing from young unimpaired to demented older adult (Fig. 6B). No significant interaction was found for PM (*F* = 1.0, *P* = 0.3).

Adjustment for FD led to similar results for both baseline and longitudinal analyses (Supplementary Table 8).

## Discussion

By analysing longitudinal data spanning from normal ageing to the full range of Alzheimer's impairments, this study offers fundamental insights into functional connectivity within medial temporal lobe networks and emphasizes dissociable effects of age and Alzheimer's disease. Through a comprehensive analysis encompassing biological and clinical perspectives, we provide compelling evidence for the relevance of these networks in understanding and tracking Alzheimer's disease.

### Distinct age-related connectivity trajectories in the AT and PM networks across the adult lifespan

Our study provides strong evidence that AT and PM undergo different ageing processes in a large sample, with longitudinal data spanning the entire adulthood. We emphasized that PM functional connectivity was reduced throughout adult life, mirroring recent findings of lower parahippocampal connectivity in relationship to age.^36^ Within AT, perirhinal connectivity was higher with anterior temporal regions and lower with regions located in frontal, parietal and posterior temporal cortices during ageing (Fig. 2). This heterogeneity might reconcile previous inconsistencies in findings reporting either higher or lower AT connectivity with older age.^38,39,41,42,59^ Prior evidence of an age-related decoupling between the default mode network and the medial temporal lobe, along with medial temporal lobe hyperconnectivity,^41,60-62^ might therefore reflect smaller-scale modifications within specialized networks.

Age effects were more pronounced for PM than for AT, which substantiates the greater functional vulnerability of posterior hippocampal and parahippocampal regions to ageing.^36,38^ This heightened sensitivity in the PM network might elucidate the cognitive impairments commonly observed in old age, particularly affecting associative memory and spatial cognition, which rely heavily on PM components.^8,9,63,64^ Vascular dysfunction is prevalent in ageing and leads to oxygen deprivation, potentially affecting the neurovascular coupling of the blood oxygen level-dependent (BOLD) signal. Thus, our findings of lower PM connectivity associated with advancing age might reflect age-related vascular changes/alterations, because the posterior part of the medial temporal lobe seems to be particularly vulnerable to vascular lesions,^65,66^ and there are significant differences in vascularization along the anteroposterior hippocampal axis.^67-69^

Interestingly, AT regions showing higher connectivity to the perirhinal cortex are particularly vulnerable to early accumulation of neurofibrillary tangles.^3,5,57,70-72^ Given that excitatory neurons are prone to tau accumulation^73,74^ and that hyperactivity can stimulate local tau secretion,^75,76^ the age-related increase in perirhinal connectivity within the AT network might be tied to primary age-related tauopathy, with hyperactivity and hyperconnectivity interplaying.^77-82^ Unfortunately, no tau data were available in our study; therefore, further research will be needed to validate this hypothesis.

### Increased AT connectivity is key to Alzheimer's disease

Analyses conducted across the Alzheimer's continuum revealed that the disease differentially affects medial temporal lobe networks, with specific disruption of AT over PM. Importantly, higher amyloid burden and lower glucose metabolism in Alzheimer-signature regions, smaller hippocampal volume, poorer cognitive performance and more advanced clinical diagnosis were consistently linked to higher AT connectivity. Although prior research on AT dysfunction in Alzheimer's disease led to inconsistent results, this new evidence advocates that AT hyperconnectivity is key in Alzheimer's pathophysiology. This substantiates previous studies describing greater functional connectivity within AT in patients with MCI or Alzheimer-dementia compared with controls,^28-30,33,34^ but also extends our knowledge by revealing that increased connectivity in the AT network is intrinsically linked to the pathological and clinical progression of the disease. Such findings align with the medial temporal lobe hyperexcitability hypothesis^12,83^ and the presence of activity-dependent degenerative processes in Alzheimer's disease.^77^ They also corroborate previous animal studies showing that hyperactivity, rather than hypoactivity, in medial temporal regions is the primary neuronal dysfunction.^84^

A speculative viewpoint is that early tau aggregates accumulate in AT hub regions. The excitatory/inhibitory imbalance caused by amyloid deposition might trigger hyperconnectivity within the AT network,^84-90^ potentially accelerating the spread of tau to PM regions via entorhinal-mediated connections.^57,91-94^ Induced neuronal toxicity would lead to hypometabolism and neurodegeneration,^25,76,95^ ultimately resulting in cognitive impairment. This metabolic imbalance, in turn, might reinforce hyperconnectivity, creating a positive feedback loop that accelerates progression of Alzheimer's.^96^ Although more insights are needed to ascertain the causal relationships underlying these circuit dysfunctions, it is evident that medial temporal functional connectivity plays a crucial role in Alzheimer-related processes. Future mechanistic studies might shed light on the associations highlighted here and enhance our understanding of the cascading processes linking hyperconnectivity in the AT network to disease propagation.

In addition, this study provides valuable insights into the prognostic significance of AT hyperconnectivity in Alzheimer's disease progression. In line with prior identification of hippocampal hyperactivity in at-risk individuals,^97^ we observed that higher AT functional connectivity was correlated with faster dementia onset in Aβ-positive MCI patients who later developed Alzheimer-dementia. These findings represent the first investigation of functional connectivity within medial temporal lobe networks in relationship to clinical evolution in prodromal patients based on longitudinal data and posit AT connectivity as a potential indicator of disease progression in symptomatic stages. Based on the aforementioned findings, it is plausible that AT hyperconnectivity, intertwined with cascading pathological events, might hasten disease advancement and ultimately precipitate dementia onset by worsening cognitive deficits.

Previous studies have robustly documented lower connectivity within the PM network in patients with MCI or Alzheimer's disease^28-30,32,98^; however, we did not observe lower PM connectivity related to any markers associated with Alzheimer pathology or clinical staging. Our approach was to focus on parahippocampal connectivity within the PM network, which might explain the discrepancies with existing findings. Indeed, reports of lower PM connectivity often encompass broader disconnections beyond our targeted core regions or/and comprised within AT mask.^28,29,59^ Further work not limited to hub regions might offer new insights into these disconnections and their associations with pathological and clinical outcomes. This drawback, however, yields more readily applicable neuroimaging biomarkers for clinical use by ensuring comparability across analyses. ROI-level analyses within PM revealed an intriguing trend: Alzheimer's features were more likely to be associated with higher parahippocampal connectivity with temporal regions of PM. These regions encompassed the entorhinal cortex, fusiform and inferior temporal gyrus. This observation suggests an ageing-opposed process, wherein temporal regions disengage to facilitate the emergence of localized hyperconnectivity, in line with the above discussion.

### Contrasting influences of ageing and Alzheimer's disease on AT/PM connectivity

Our analyses across the entire sample well illustrated the effects of age and Alzheimer's disease on hub functional connectivity in medial temporal lobe networks. Although reduced PM connectivity appears to result from normal ageing, Alzheimer's disease is characterized by AT hyperconnectivity, with subtle increased connectivity already observed in specific regions of the network during ageing.

Previous animal research indicates that the early stages of Alzheimer's disease are characterized by neuronal hyperactivity, succeeded by neuronal silencing in later stages.^99^ This notion finds support in a recent neuroimaging investigation focusing on medial temporal lobe functional connectivity, which demonstrated higher connectivity within AT regions during preclinical phases of Alzheimer's disease, but reduced connectivity in advanced stages.^36^ Despite modelling our data without *a priori* inferences, we found no evidence for such an inverted U-shaped trajectory across disease stages (Fig. 6A). However, it is worth noting that our study included patients with only mild to moderate Alzheimer-dementia, and exploration of severe dementia cases might unveil late AT outcomes. Compared with the non-ranking approach (Fig. 3B), classifying groups across the entire sample according to hierarchical order of the adult lifespan and the Alzheimer's continuum (Fig. 6B) highlighted that AT connectivity showed an accelerated rate of increase over time. This suggests a worsening of AT hyperconnectivity-related consequences with progression of Alzheimer's disease, following the previously hypothesized trajectory.^54-56,100^

For the PM network, we observed distinct changes during normal ageing and Alzheimer's disease. Consistent with our previous findings, lower connectivity within PM was found specifically to reflect age-related processes. In contrast, the slight increase in PM connectivity across the Alzheimer's continuum (Fig. 6A) might reflect Alzheimer-featured parahippocampal hyperconnectivity, particularly within the temporal regions of the PM network (Fig. 4B).

Of note, it is important to clarify that when we refer to AT or PM connectivity in our study, we specifically mean perirhinal connectivity within the AT network or parahippocampal connectivity within the PM network. This seed-based approach might explain differences from previous studies and underscores the specific contributions of hub regions within medial temporal lobe functional networks in ageing and Alzheimer's disease. Consequently, the increased AT connectivity observed in our results is likely to reflect changes in the perirhinal cortex associated with the pathological and clinical progression of Alzheimer's disease.

Overall, these findings support the theory that early Alzheimer's changes weaken distant connections and strengthen proximal ones.^12^ Given that prior research indicated reduced AT and PM network specialization in older adults attributable to Alzheimer's pathology,^26^ we propose that medial temporal lobe networks operate independently in early adulthood and undergo network dedifferentiation throughout the progression of Alzheimer's disease.

### Strengths and limitations

The main strengths of this study include the monocentric dataset with available longitudinal (f)MRI, amyloid- and ^18^F-fluorodeoxyglucose-PET, clinical and cognitive data, which allowed us to estimate various features of Alzheimer's disease. In addition, we opted for a refined methodology reaching for individual-level optimal seeds to ensure accuracy and replicability of networks. Another key point is the development of easier-to-interpret fMRI outcomes, conceived as standardized indexes to provide global proxies for network functioning that might estimate the risk of disease progression if further validation steps are met. Nevertheless, our design only counts a limited follow-up and small sample size in some groups. Specifically, a larger pool of Aβ-positive cognitively unimpaired older adults is crucial to draw conclusions about early diagnosis, because only eight participants were classified as preclinical Alzheimer's disease, hence no robust conclusions can be drawn from this sample. This is particularly important because other studies have already pointed to differences at this stage,^29,59^ and our analyses of Alzheimer's neuroimaging features endorse the importance of AT functional connectivity in early Alzheimer-related brain changes. Longer follow-up might also provide more sensitivity to our analyses, especially with respect to changes over time that might be undetectable with a limited number of scans. The lack of tau data is another important limitation in interpreting our results. Particularly, future studies incorporating tau-PET would help to elucidate further the relationships between functional alterations within the AT and PM networks and the accumulation of Alzheimer's pathologies.

## Conclusion

The present longitudinal study yields key insights into functional connectivity within medial temporal lobe networks across physiological ageing and the Alzheimer's disease continuum. Our results clearly identified Alzheimer's signature connectivity changes, showing that all biomarkers related to Alzheimer's pathology or clinical staging were consistently associated with AT hyperconnectivity. This network alteration warrants close attention because it might catalyse disease progression according to connectome-based propagation models. Future research into the cascading events leading to lesion propagation, particularly regarding AT/PM connectivity, might enhance our understanding of Alzheimer's pathophysiology and hold promise for the development of prognostic tools and network-targeted therapeutic interventions.

## Supplementary Material

awaf008_Supplementary_Data

## Data Availability

The dataset used in the present study is available from G.C. upon reasonable request, for the purpose of replicating procedures and results. The codes used for data analyses can be requested from L.C. or R.d.F.
