# Supplementary material for: Anterior-temporal network hyperconnectivity is key to Alzheimer’s disease: from ageing to dementia
Source: Brain. 2025 Jan 15;148(6):2008–22. doi: 10.1093/brain/awaf008 (PMC12129740; doi:10.1093/brain/awaf008)
Supplement: awaf008_Supplementary_Data [file awaf008_supplementary_data.pdf]

Supplementary Materials

Table of contents

Supplementary Methods 2
Supplementary Tables 3
Supplementary Table 1. 3
Supplementary Table 2. 9
Supplementary Table 3. 16
Supplementary Table 4. 20
Supplementary Table 5. 26
Supplementary Table 6.. 26
Supplementary Table 7. 27
Supplementary Table 8. 27
Supplementary Table 9. 28
Supplementary Table 10. 29
Supplementary Table 11. 30
Supplementary Table 12. 30
Supplementary Table 13. 31
Supplementary Table 14. 32
Supplementary Table 15. 33
Supplementary Table 16. 34
Supplementary Table 17. 35
Supplementary Table 18. 36
Supplementary Table 19. 37
Supplementary Table 20. 38
Supplementary Table 21. 39
Supplementary Table 22. 40
Supplementary Figures 42
Supplementary Figure 1. 42
Supplementary Figure 2. 42
Supplementary Figure 3. 43
Supplementary Figure 4. 43
Supplementary Figure 5. 44
Supplementary Figure 6. 44
Supplementary Figure 7. 45
Supplementary Figure 8. 45
Supplementary Figure 9. 46
Supplementary Figure 10. 46
Supplementary Figure 11. 47

Supplementary Methods

Assessment of cognitive performance in healthy individuals

A comprehensive battery of conventional and validated tests was administered to healthy volunteers to ensure that their cognitive performances were within the standard range across multiple domains, encompassing global cognitive functioning, depressive symptoms, language abilities, episodic verbal and visual memory, executive functions, visuospatial abilities, and gestural praxis. The tests included the mini-mental state examination (MMSE; Folstein et al. 1975), the Mattis dementia rating scale (MDRS; Mattis, 1976), the Montgomery and Asberg depression rating scale (Montgomery & Asberg, 1979), the Mill Hill vocabulary scale (Oldfield, 1971), a picture naming test (DO-80, Deloche & Hannequin, 1997), a writing task involving 12 irregular words, two verbal fluency tests (Cardebat et al. 1990), Grober & Buschke's test (1987), recall of the BEM 144 figure (Signoret et al., 1991), Stroop test (1935), Rey figure copying (1959), imitation of four meaningless gestures, and production of four symbolic gestures and four gestures related to object use (Mahieux et al. 2009).

Supplementary Tables

<u>Supplementary Table 1.</u> Glossary of regions within the anterior-temporal (AT) mask of young adults (<40 years old) derived from the Brainnetome atlas.

Region code	Brainnetome atlas labelling	Brain view
BN_AT_IPL_A39c	Inferior parietal lobule – caudal area 39	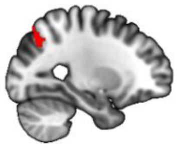
BN_AT_LOcC_lsOccG	Lateral occipital cortex - lateral superior occipital gyrus	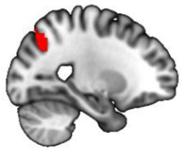
BN_AT_IFG_IFS	Inferior frontal gyrus – inferior frontal sulcus	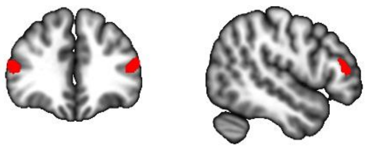
BN_AT_IPL_A39rd	Inferior parietal lobule - rostrodorsal area 39	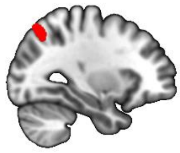
BN_AT_SPL_A7ip	Superior parietal lobule - intraparietal area 7	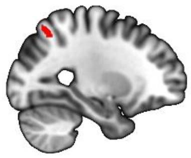
BN_AT_SPL_A7c	Superior parietal lobule - caudal area 7	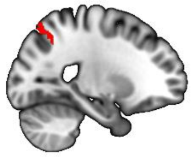
BN_AT_ITG_A37vl	Inferior temporal gyrus - ventrolateral area 37	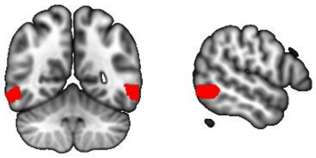

BN_AT_ITG_A37elv	Inferior temporal gyrus - extreme lateroventral area 37	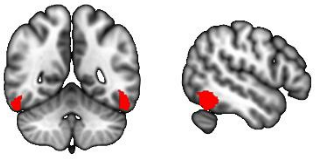
BN_AT_FuG_A37lv	Fusiform gyrus - lateroventral area 37	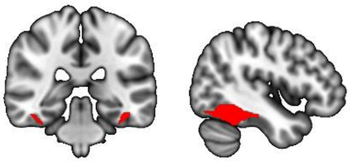
BN_AT_MTG_A37dl	Middle temporal gyrus - dorsolateral area 37	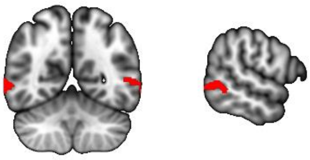
BN_AT_PhG_TL	Parahippocampal gyrus - area TL (lateral posterior parahippocampal gyrus)	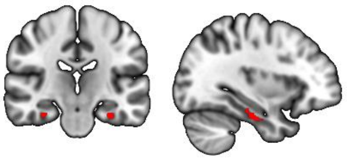
BN_AT_OrG_A12_47o	Orbital gyrus - orbital area 12/47	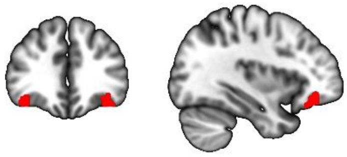
BN_AT_ITG_A20cl	Inferior temporal gyrus - caudolateral of area 20	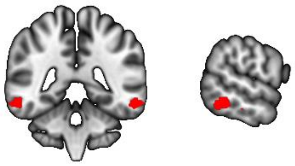
BN_AT_OrG_A11l	Orbital gyrus - lateral area 11	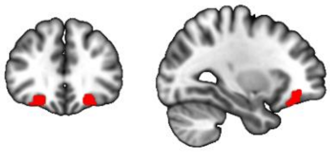

BN_AT_LOcC_V5_MT	Lateral occipital cortex - area V5/MT+	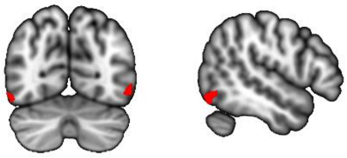
BN_AT_Tha_rTtha	Thalamus - rostral temporal thalamus	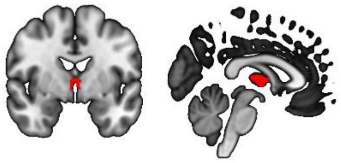
BN_AT_MTG_A21c	Middle temporal gyrus - caudal area 21	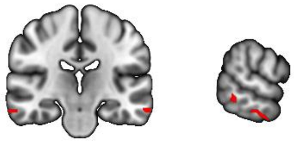
BN_AT_Amyg_mAmyg	Amygdala - medial amygdala	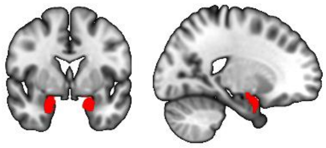
BN_AT_OrG_A13	Orbital gyrus – area 13	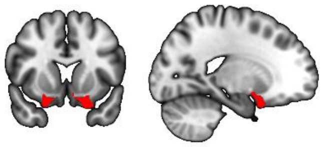
BN_AT_STG_A38l	Superior temporal gyrus - lateral area 38	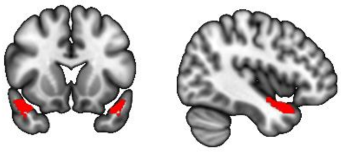
BN_AT_Tha_mPFtha	Thalamus - medial pre-frontal thalamus	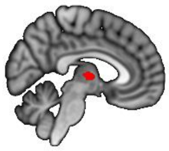

BN_AT_STG_A22r	Superior temporal gyrus - rostral area 22	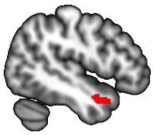
BN_AT_PhG_A35_36c	Parahippocampal gyrus - caudal area 35/36	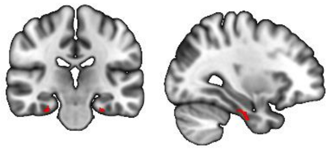
BN_AT_BG_NAC	Basal ganglia – nucleus accumbens	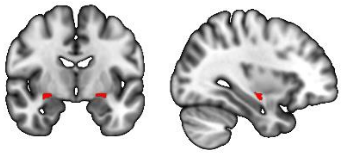
BN_AT_ITG_A20cv	Inferior temporal gyrus - caudoventral of area 20	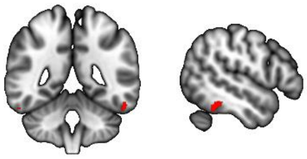
BN_AT_BG_dlPu	Basal ganglia - dorsolateral putamen	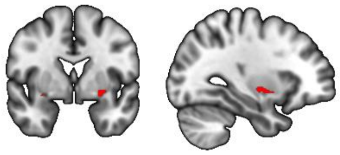
BN_AT_MTG_A21r	Middle temporal gyrus - rostral area 21	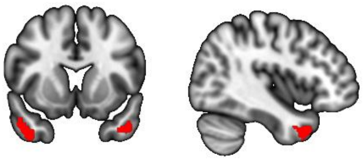
BN_AT_Amyg_lAmyg	Amygdala - lateral amygdala	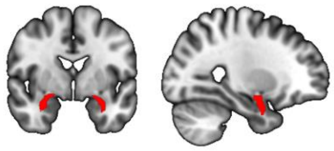

BN_AT_BG_vmPu	Basal ganglia - ventromedial putamen	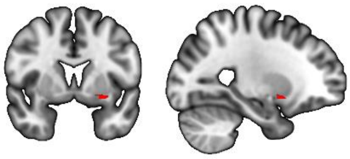
BN_AT_STG_TE10_TE12	Superior temporal gyrus - TE1.0 and TE1.2	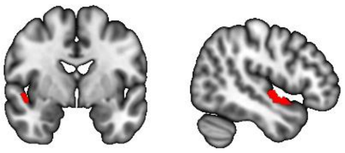
BN_AT_INS_vld_vlg	Insular gyrus - ventral dysgranular and granular insula	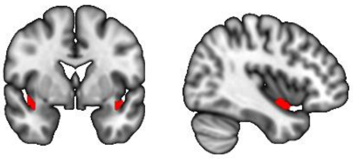
BN_AT_INS_vla	Insular gyrus - ventral agranular insula	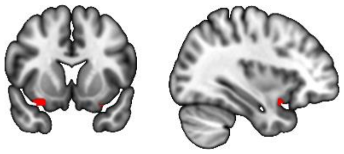
BN_AT_ITG_A20il	Inferior temporal gyrus - intermediate lateral area 20	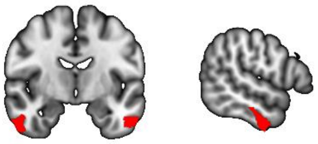
BN_AT_Hipp_rHipp	Hippocampus- rostral hippocampus	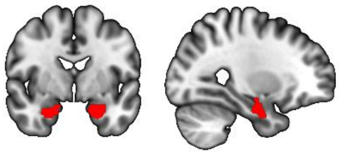
BN_AT_Hipp_cHipp	Hippocampus- caudal hippocampus	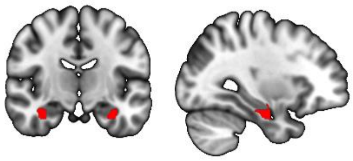

BN_AT_ITG_A20iv	Inferior temporal gyrus - intermediate ventral area 20	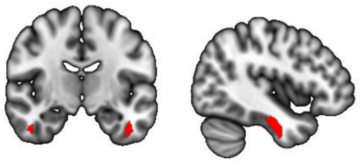
BN_AT_ITG_A20r	Inferior temporal gyrus - rostral area 20	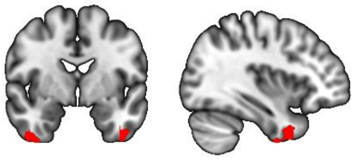
BN_AT_STG_A38m	Superior temporal gyrus - medial area 38	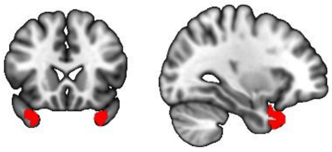
BN_AT_FuG_A20rv	Fusiform gyrus - rostroventral area 20	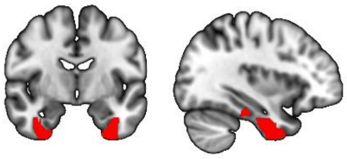
BN_AT_PhG_A35_36r	Parahippocampal gyrus - rostral area 35/36	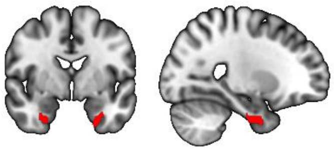
BN_AT_PhG_TI	Parahippocampal gyrus - area TI (temporal agranular insular cortex)	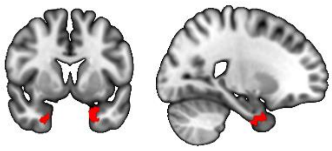
BN_AT_PhG_A28_34	Parahippocampal gyrus - area 28/34 (entorhinal cortex)	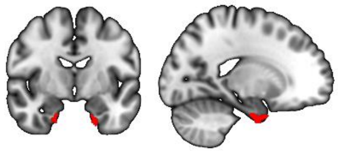

Supplementary Table 2. Glossary of regions within the posterior-medial (PM) mask of young adults (<40 years old) derived from the Brainnetome atlas.

Region code	Brainnetome atlas labelling	Brain view
BN_PM_PhG_TL	Parahippocampal gyrus - area TL (lateral posterior parahippocampal gyrus)	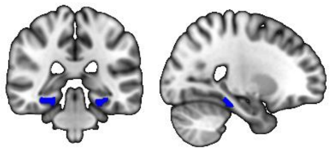
BN_PM_FuG_A20rv	Fusiform gyrus - rostroventral area 20	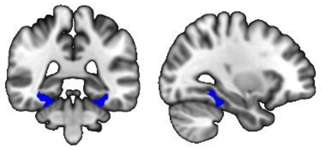
BN_PM_PhG_TH	Parahippocampal gyrus - area TH (medial posterior parahippocampal gyrus)	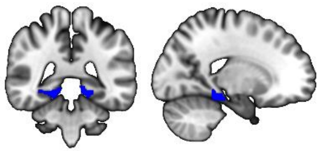
BN_PM_PCun_dmPOS	Precuneus - dorsomedial parietooccipital sulcus	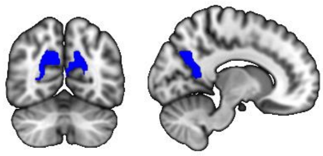
BN_PM_CG_A23v	Cingulate gyrus - ventral area 23	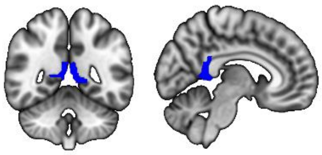
BN_PM_OrG_A14m	Orbital gyrus - medial area 14	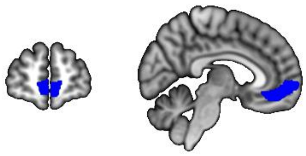
BN_PM_PCun_A31	Precuneus - area 31	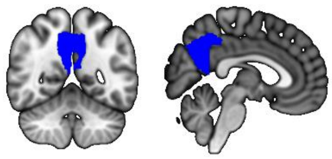

BN_PM_SFG_A8dl	Superior frontal gyrus - dorsolateral area 8	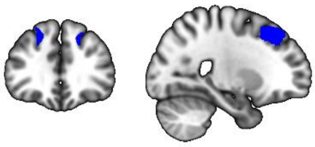
BN_PM_MFG_A9_46d	Middle frontal gyrus - dorsal area 9/46	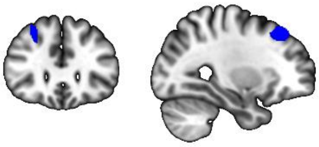
BN_PM_MFG_A8vl	Middle frontal gyrus - ventrolateral area 8	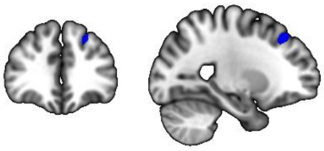
BN_PM_IPL_A39rv	Inferior parietal lobule - rostroventral area 39	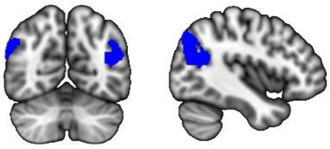
BN_PM_IPL_A39rd	Inferior parietal lobule - rostrodorsal area 39	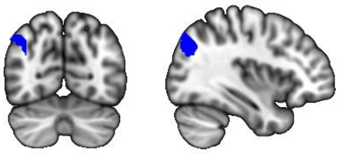
BN_PM_OrG_A11m	Orbital gyrus – medial area 11	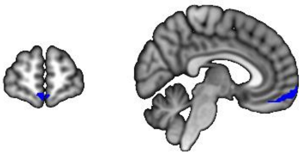
BN_PM_CG_A23c	Cingulate gyrus - caudal area 23	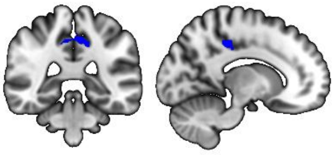
BN_PM_IPL_A39c	Inferior parietal lobule - caudal area 39	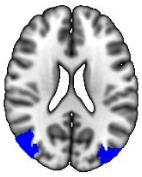

BN_PM_CG_A23d	Cingulate gyrus - dorsal area 23	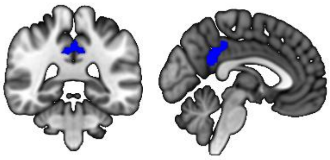
BN_PM_Hipp_rHipp	Hippocampus – rostral hippocampus	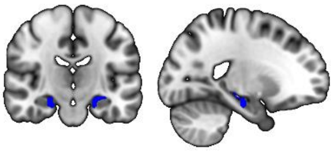
BN_PM_PhG_A28_34	Parahippocampal gyrus - area 28/34 (entorhinal cortex)	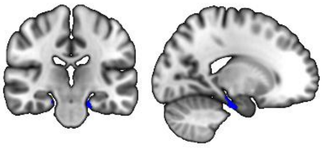
BN_PM_OrG_A13	Orbital gyrus - area 13	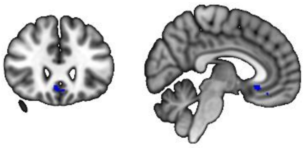
BN_PM_PCun_A7m	Precuneus - medial area 7	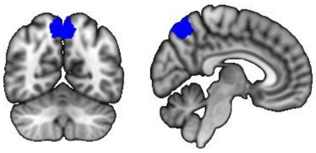
BN_PM_PhG_A35_36c	Parahippocampal gyrus - caudal area 35/36	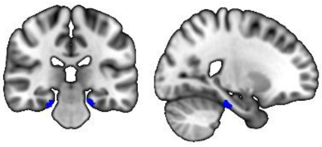
BN_PM_SFG_A10m	Superior frontal gyrus - medial area 10	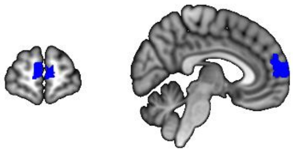
BN_PM_MVOcC_rLinG	Medio ventral occipital cortex - rostral lingual gyrus	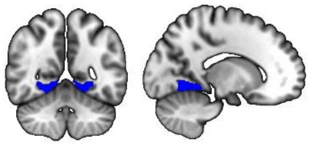

BN_PM_MVOcC_vmPOS	Medio ventral occipital cortex - ventromedial parietooccipital sulcus	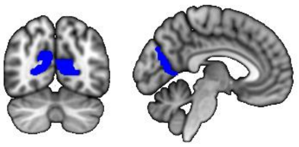
BN_PM_MFG_A6vl	Middle frontal gyrus - ventrolateral area 6	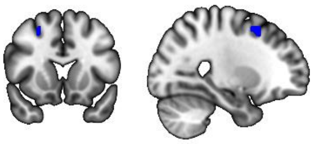
BN_PM_PCun_A5m	Precuneus - medial area 5	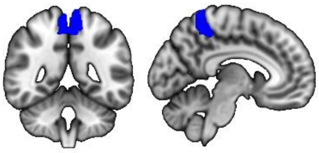
BN_PM_MTG_A37dl	Middle temporal gyrus - dorsolateral area 37	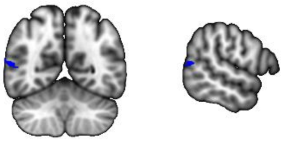
BN_PM_SPL_A7r	Superior parietal lobule - rostral area 7	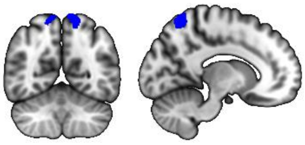
BN_PM_CG_A32sg	Cingulate gyrus - subgenual area 32	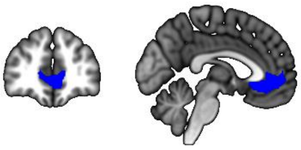
BN_PM_CG_A24rv	Cingulate gyrus - rostroventral area 24	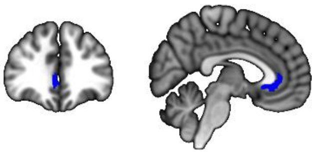
BN_PM_FuG_A37mv	Fusiform gyrus - medioventral area 37	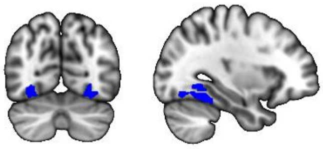

BN_PM_Tha_Otha	Thalamus – occipital thalamus	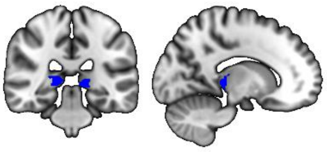
BN_PM_SFG_A9m	Superior frontal gyrus - medial area 9	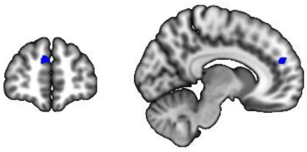
BN_PM_LOcC_lsOccG	Lateral occipital cortex - lateral superior occipital gyrus	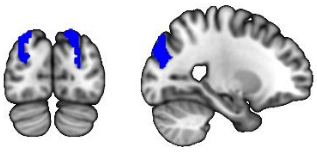
BN_PM_Tha_cTtha	Thalamus - caudal temporal thalamus	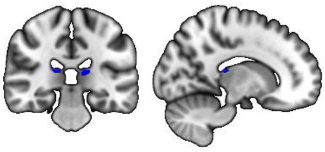
BN_PM_Hipp_cHipp	Hippocampus - caudal hippocampus	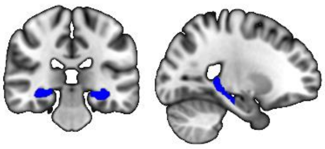
BN_PM_LOcC_msOccG	Lateral occipital cortex - medial superior occipital gyrus	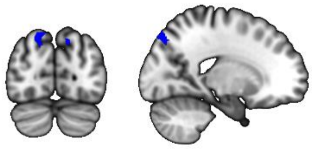
BN_PM_Tha_PPtha	Thalamus - posterior parietal thalamus	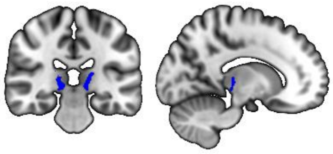
BN_PM_FuG_A37lv	Fusiform gyrus - lateroventral area 37	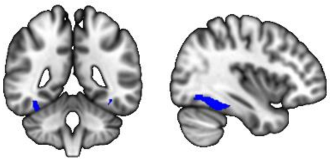

BN_PM_SPL_A7c	Superior parietal lobule - caudal area 7	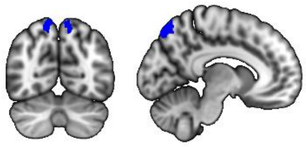
BN_PM_PCL_A1_2_3ll	Paracentral lobule – area 1/2/3 (lower limb region)	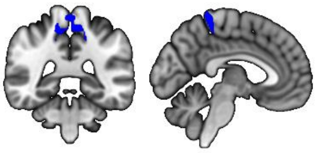
BN_PM_MVOcC_rCunG	Medioventral occipital cortex - rostral cuneus gyrus	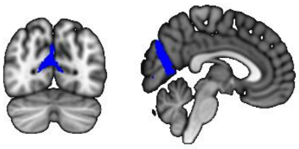
BN_PM_LOcC_mOccG	Lateral occipital cortex – middle occipital gyrus	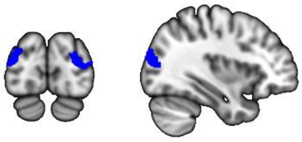
BN_PM_LOcC_V5_MT	Lateral occipital cortex - area V5/MT+	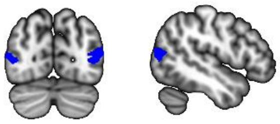
BN_PM_LOcC_OPC	Lateral occipital cortex - occipital polar cortex	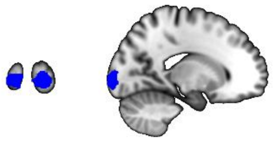
BN_PM_MVOcC_cLinG	Medioventral occipital cortex - caudal lingual gyrus	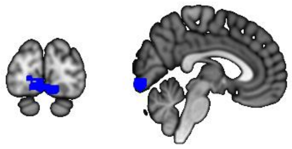
BN_PM_MVOcC_cCunG	Medioventral occipital cortex - caudal cuneus gyrus	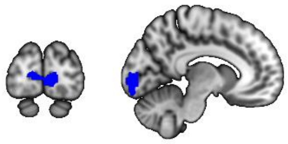

BN_PM_LOcC_iOccG	Lateral occipital cortex - inferior occipital gyrus	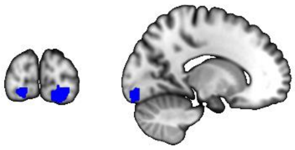

<u>Supplementary Table 3.</u> Glossary of regions within the anterior-temporal (AT) mask of Aβ-negative older adults (>60 years old) derived from the Brainnetome atlas.

Region code	Brainnetome atlas labelling	Brain view
BN_AT_ITG_A20il	Inferior temporal gyrus - intermediate lateral area 20	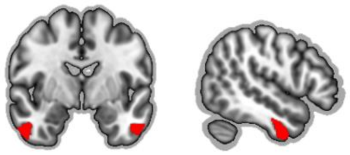
BN_AT_ITG_A20iv	Inferior temporal gyrus - intermediate ventral area 20	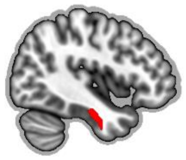
BN_AT_ITG_A20r	Inferior temporal gyrus - rostral area 20	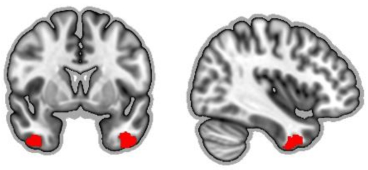
BN_AT_STG_TE10_TE12	Superior temporal gyrus - TE1.0 and TE1.2	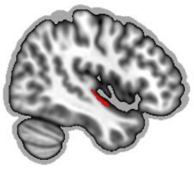
BN_AT_MTG_A21r	Middle temporal gyrus - rostral area 21	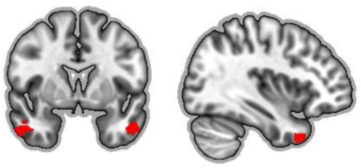
BN_AT_Hipp_cHipp	Caudal hippocampus	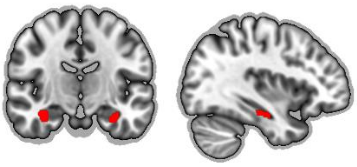
BN_AT_FuG_A20rv	Fusiform gyrus - rostroventral area 20	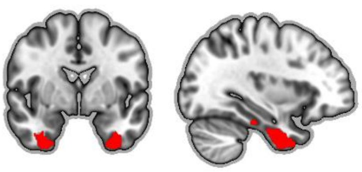

BN_AT_PhG_A28_34	Parahippocampal gyrus - entorhinal cortex	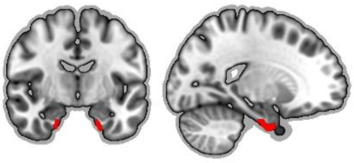
BN_AT_PhG_TL	Parahippocampal gyrus – lateral posterior parahippocampal gyrus	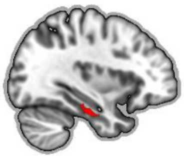
BN_AT_PhG_A35_36r	Parahippocampal gyrus - rostral area 35/36	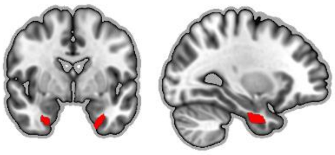
BN_AT_Hipp_rHipp	Rostral hippocampus	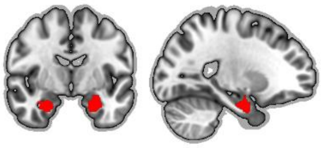
BN_AT_STG_A38l	Superior temporal gyrus - lateral area 38	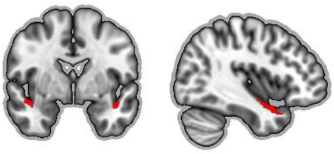
BN_AT_INS_vId_vIg	Ventral dysgranular and granular insula	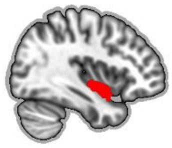
BN_AT_PhG_TI	Parahippocampal gyrus - temporal agranular insular cortex	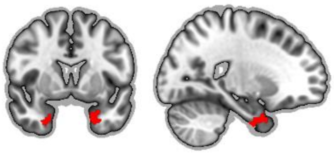
BN_AT_STG_A38m	Superior temporal gyrus - medial area 38	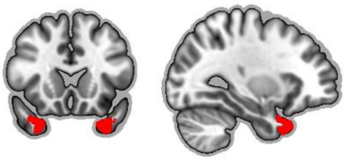

BN_AT_Amyg_mAmyg	Medial amygdala	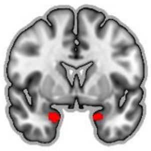
BN_AT_PhG_A35_36c	Parahippocampal gyrus - caudal area 35/36	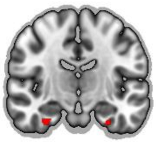
BN_AT_Amyg_lAmyg	Lateral amygdala	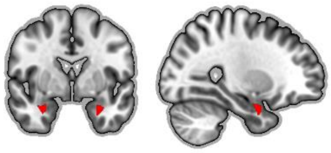
BN_AT_BG_NAC	Nucleus accumbens	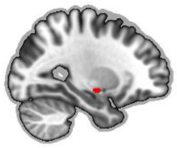
BN_AT_OrG_A12_47o	Orbital gyrus - orbital area 12/47	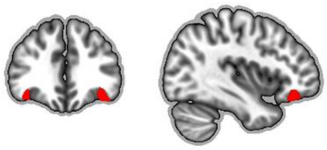
BN_AT_ITG_A37elv	Inferior temporal gyrus - extreme lateroventral area 37	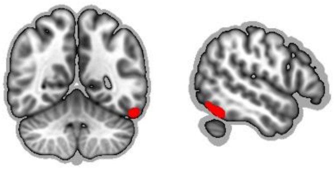
BN_AT_OrG_A11l	Orbital gyrus - lateral area 11	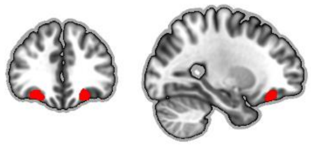
BN_AT_ITG_A37vl	Inferior temporal gyrus - ventrolateral area 37	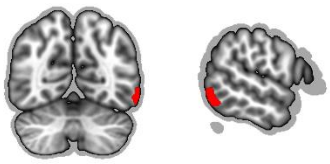

BN_AT_FuG_A37lv	Fusiform gyrus - lateroventral area 37	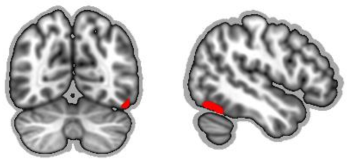

<u>Supplementary Table 4.</u> Glossary of regions within the posterior-medial (PM) mask of Aβ-negative older adults (>60 years old) derived from the Brainnetome atlas.

Region code	Brainnetome atlas labelling	Brain view
BN_PM_PhG_A35_36c	Parahippocampal gyrus - caudal area 35/36	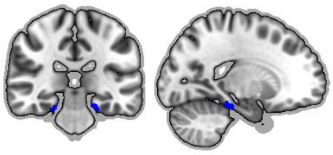
BN_PM_FuG_A37mv	Fusiform gyrus - medioventral area37	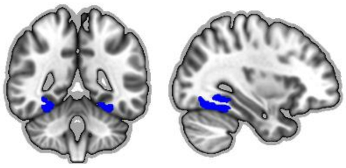
BN_PM_MVOcC_rLinG	Medio ventral occipital cortex - rostral lingual gyrus	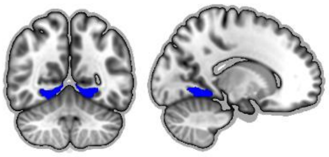
BN_PM_LOcC_iOccG	Lateral occipital cortex - inferior occipital gyrus	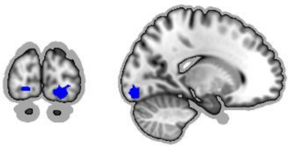
BN_PM_Tha_Otha	Occipital thalamus	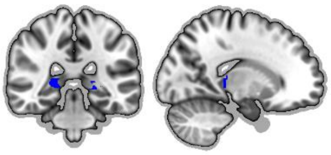
BN_PM_PhG_TH	Parahippocampal gyrus - medial posterior parahippocampal gyrus	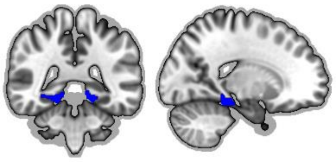
BN_PM_FuG_A37lv	Fusiform gyrus - lateroventral area 37	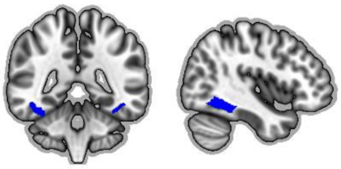

BN_PM_MVOcC_cLinG	Medio-ventral occipital cortex - caudal lingual gyrus	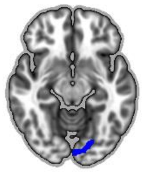
BN_PM_Hipp_cHipp	Caudal hippocampus	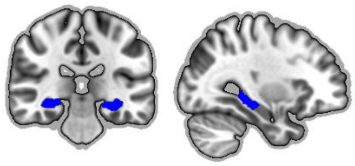
BN_PM_LOcC_OPC	Lateral occipital cortex - occipital polar cortex	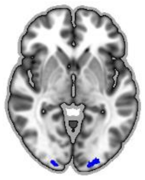
BN_PM_FuG_A20rv	Fusiform gyrus - rostroventral area 20	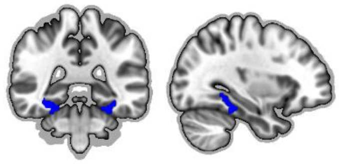
BN_PM_MVOcC_cCunG	Medio-ventral occipital cortex - caudal cuneus gyrus	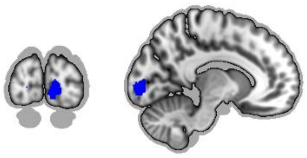
BN_PM_LOcC_mOccG	Lateral occipital cortex - medial superior occipital gyrus	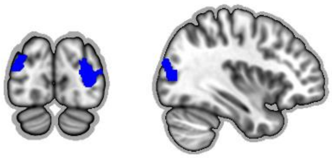
BN_PM_PhG_TL	Parahippocampal gyrus - lateral posterior parahippocampal gyrus	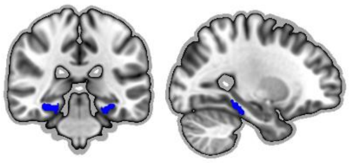

BN_PM_ITG_A37vl	Inferior temporal gyrus - ventrolateral area 37	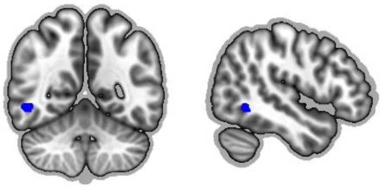
BN_PM_Hipp_rHipp	Rostral hippocampus	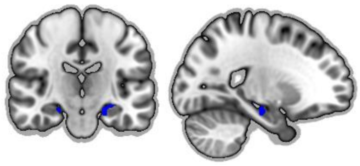
BN_PM_LOcC_V5_MT	Lateral occipital cortex - area V5/MT+	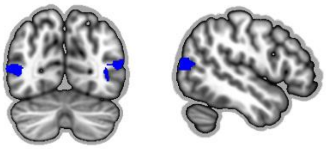
BN_PM_PCL_A1_2_3ll	Paracentral lobule - area1/2/3 (lower limb region)	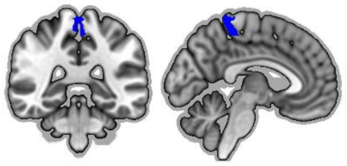
BN_PM_OrG_A11m	Orbital gyrus - medial area 11	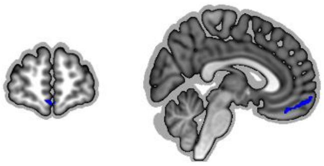
BN_PM_MVOcC_rCunG	Medio-ventral occipital cortex - rostral cuneus gyrus	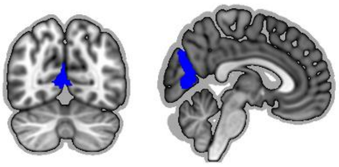
BN_PM_PCL_A4ll	Paracentral lobule - area 4 (lower limb region)	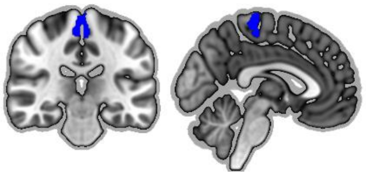

BN_PM_CG_A23v	Cingulate gyrus - ventral area 23	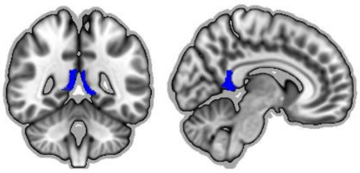
BN_PM_CG_A32sg	Cingulate gyrus - subgenual area 32	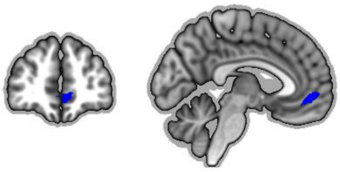
BN_PM_PCun_A31	Precuneus - area 31 (Lc1)	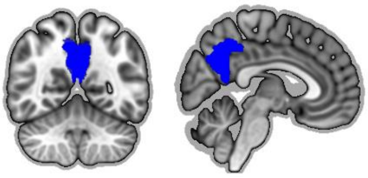
BN_PM_LOcC_lsOccG	Lateral occipital cortex - lateral superior occipital gyrus	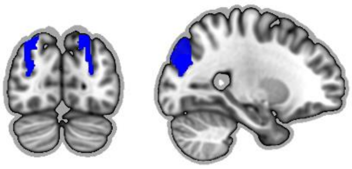
BN_PM_PCun_A5m	Precuneus - medial area 5(PEm)	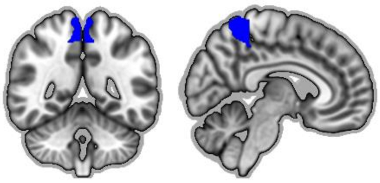
BN_PM_LOcC_msOccG	Lateral occipital cortex - medial superior occipital gyrus	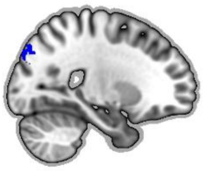
BN_PM_OrG_A14m	Orbital gyrus - medial area 14	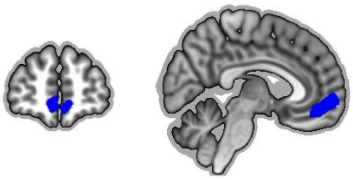

BN_PM_CG_A23d	Cingulate gyrus - dorsal area 23	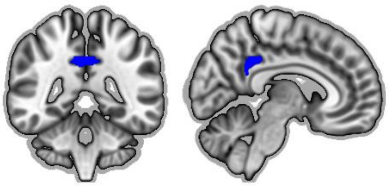
BN_PM_IPL_A39c	Inferior parietal lobule - caudal area 39 (PGp)	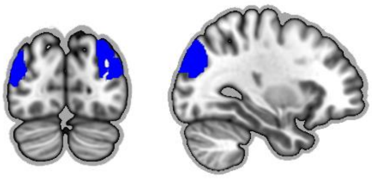
BN_PM_MVOcC_vmPOS	Medio-ventral occipital cortex - ventromedial parietooccipital sulcus	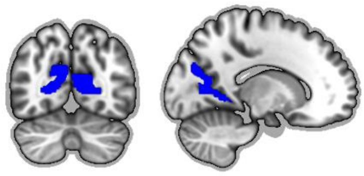
BN_PM_SPL_A7r	Superior parietal lobule - rostral area 7	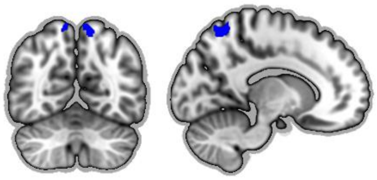
BN_PM_MFG_A6vl	Middle frontal gyrus - ventrolateral area 6	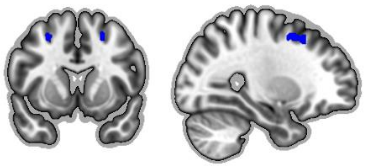
BN_PM_IPL_A39rv	Inferior parietal lobule - rostroventral area 39 (PGa)	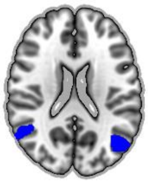
BN_PM_MFG_A9_46d	Middle frontal gyrus - dorsal area 9/46	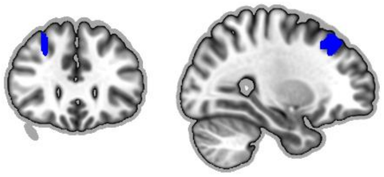

BN_PM_PCun_A7m	Precuneus - medial area 7 (PEp)	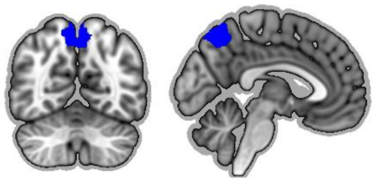
BN_PM_PCun_dmPOS	Precuneus - dorsomedial parietooccipital sulcus (PEr)	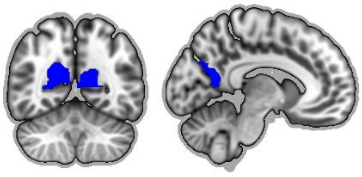
BN_PM_MFG_A8vl	Middle frontal gyrus - ventrolateral area 8	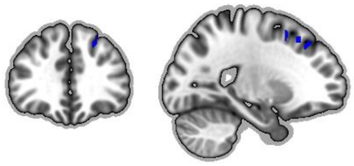
BN_PM_IPL_A39rd	Inferior parietal lobule - rostrodorsal area 39 (Hip3)	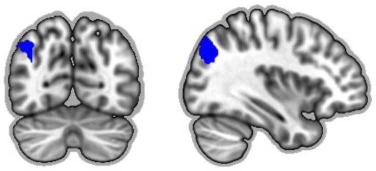
BN_PM_SFG_A8dl	Superior frontal gyrus - dorsolateral area 8	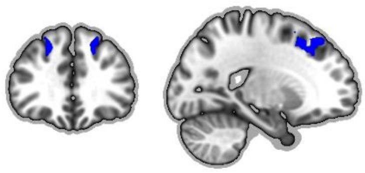
BN_PM_SPL_A7c	Superior parietal lobule - caudal area 7	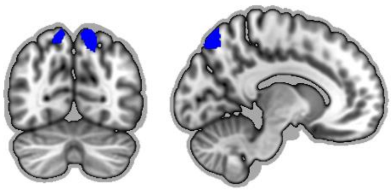

Supplementary Table 5. Replication analyses using masks of young adults (<40 years old). Statistical summary of functional connectivity in the anterior-temporal (AT) and posterior-medial (PM) networks across the whole sample.

	edf	*F*	*P*
Group			
AT	1.56	13.00	**<0.001**
PM	2.27	9.28	**<0.001**
Time * Group			
AT	310.13	4.64	**0.03**
PM	325.87	1.03	0.31

The *group* effects, ranging from cognitively unimpaired young adults to older adults with Alzheimer's disease, were derived from generalized additive models fitted to baseline data. Visual inspection of the smooth term indicates similar trajectories to those illustrated in Fig. 6A. The *time* x *group* interaction effects were derived from linear mixed models fitted to longitudinal data. Visual inspection of post-hoc predictions indicates similar effects to those depicted in Fig. 6B.

Supplementary Table 6. Replication analyses modelling connectivity of the anterior-temporal (AT) and posterior-medial (PM) networks simultaneously. Statistical summary of the main analyses of the study.

	**Interaction term**			**Post-hoc**			
	ddf	*F*	*P*		trend	*t*	*P*
Age	582.2	30.2	**<0.001**	AT	0.0002	1.7	0.097
				PM	-0.0007	-4.7	**<0.001**
Amyloid uptake	304.8	8.6	**0.004**	AT	0.1620	4.8	**<0.001**
				PM	0.0386	1.1	0.26
Glucose metabolism	328.7	15.3	**<0.001**	AT	-0.1070	-6.5	**<0.001**
				PM	-0.0254	-1.5	0.13
Hippocampal volume	356.6	25.9	**<0.001**	AT	-0.0864	-5.1	**<0.001**
				PM	0.0121	0.7	0.47
MMSE	359.5	5.3	**0.02**	AT	-0.0050	-4.4	**<0.001**
				PM	-0.0018	-1.6	0.11
MDRS	334.5	8.0	**0.005**	AT	-0.0019	-4.5	**<0.001**
				PM	-0.0004	-1.0	0.33
MCI-to-AD time	69	13.0	**<0.001**	AT	-0.0156	-3.3	**0.002**
				PM	0.0071	1.5	0.14

All outputs were consistent with statistics reported in Results, except for the effects of age on AT connectivity that was not robust across both approaches.

Supplementary Table 7. Summary of framewise displacement among study groups.

	**Unimpaired young & middle-aged adults**	**Unimpaired older adults**	**MCI patients**	**AD-demented patients**
**Framewise displacement**	0.05 ± 0.04 [0.01, 0.26]	0.07 ± 0.04 [0.01, 0.36]	0.1 ± 0.05 [0.02, 0.21]	0.09 ± 0.06 [0.03, 0.29]

Supplementary Table 8. Replication analyses including Framewise displacement (FD) as a covariate. **P*-values after the Holm-Bonferroni's controlling procedure.

	Network	**β**	***F***	***P***
**Age-related trajectories**				
	AT	0.0000	0.0	0.9
	PM	-0.0008	27.2	**<0.001**
**Differences among Alzheimer's stages**				
**Baseline**	AT		2.1	0.1
	PM		0.4	0.7
**Associations with Alzheimer's disease neuroimaging biomarkers and global cognition**				
**Amyloid uptake**	AT	0.1344	13.0	**0.003***
	PM	0.0357	1.4	0.7*
**Glucose metabolism**	AT	-0.0881	23.4	**<0.001***
	PM	-0.0307	4.3	0.2*
**Hippocampal volume**	AT	-0.0519	7.3	**0.007***
	PM	-0.0010	0.0	0.9*
**MMSE**	AT	-0.0037	9.4	**0.007***
	PM	-0.0018	3.4	0.3*
**MDRS**	AT	-0.0015	11.0	**0.005***
	PM	-0.0004	1.3	0.7*
**Association with delay to Alzheimer-dementia onset (in MCI converters)**				
	AT	-0.0101	2.8	***0.05***
	PM	0.00787	2.4	0.1
**Difference across the whole sample**				
**Baseline**	AT		7.9	**<0.001**
	PM		10.1	**<0.001**
**Over time**	AT		6.1	**0.01**
	PM		1.1	0.3

Supplementary Table 9. Age-related perirhinal connectivity with subregions of the anterior-temporal (AT) network.

	ddf	*F*	trend	*P*	adjusted *P*
BN_AT_IPL_A39c	173.83	27.42	-0.0014	0.000	**0.000**
BN_AT_IFG_IFS	182.23	22.23	-0.0013	0.000	**0.000**
BN_AT_LOcC_lsOccG	176.72	26.44	-0.0013	0.000	**0.000**
BN_AT_IPL_A39rd	178.23	26.81	-0.0012	0.000	**0.000**
BN_AT_SPL_A7ip	176.31	21.52	-0.0011	0.000	**0.000**
BN_AT_SPL_A7c	174.27	20.40	-0.0010	0.000	**0.000**
BN_AT_ITG_A37elv	175.03	17.19	-0.0010	0.000	**0.002**
BN_AT_ITG_A37vl	176.82	19.31	-0.0010	0.000	**0.001**
BN_AT_FuG_A37lv	174.74	16.71	-0.0009	0.000	**0.002**
BN_AT_MTG_A37dl	160.69	16.13	-0.0008	0.000	**0.002**
BN_AT_OrG_A12_47o	173.07	7.68	-0.0006	0.006	0.130
BN_AT_PhG_TL	182.06	2.96	-0.0006	0.087	1.000
BN_AT_ITG_A20cl	165.28	9.07	-0.0006	0.003	0.072
BN_AT_LOcC_V5_MT	169.81	3.37	-0.0004	0.068	1.000
BN_AT_OrG_A11l	185.82	3.52	-0.0004	0.062	1.000
BN_AT_Tha_rTtha	172.48	2.63	-0.0003	0.107	1.000
BN_AT_MTG_A21c	177.64	1.31	-0.0002	0.254	1.000
BN_AT_Amyg_mAmyg	170.16	0.37	-0.0001	0.543	1.000
BN_AT_OrG_A13	176.91	0.29	-0.0001	0.590	1.000
BN_AT_STG_A38l	181.72	0.04	0.0000	0.833	1.000
BN_AT_STG_A22r	184.22	0.21	0.0001	0.645	1.000
BN_AT_Tha_mPFtha	179.70	0.33	0.0001	0.564	1.000
BN_AT_BG_NAC	162.31	0.63	0.0001	0.429	1.000
BN_AT_BG_dlPu	165.05	0.99	0.0002	0.321	1.000
BN_AT_MTG_A21r	180.39	0.74	0.0002	0.390	1.000
BN_AT_Amyg_lAmyg	182.17	0.85	0.0002	0.359	1.000
BN_AT_PhG_A35_36c	188.21	0.23	0.0002	0.630	1.000
BN_AT_ITG_A20cv	193.77	0.68	0.0002	0.411	1.000
BN_AT_BG_vmPu	168.46	2.15	0.0002	0.145	1.000
BN_AT_INS_vId_vIg	177.51	2.61	0.0003	0.108	1.000
BN_AT_INS_vIa	170.97	3.02	0.0004	0.084	1.000
BN_AT_STG_TE10_TE12	165.61	4.61	0.0004	0.033	0.666
BN_AT_ITG_A20il	185.95	7.77	0.0006	0.006	0.129
BN_AT_Hipp_rHipp	179.53	8.71	0.0007	0.004	0.083
BN_AT_Hipp_cHipp	169.99	12.88	0.0009	0.000	**0.011**
BN_AT_ITG_A20r	184.22	19.61	0.0010	0.000	**0.001**
BN_AT_ITG_A20iv	192.49	12.05	0.0010	0.001	**0.016**
BN_AT_STG_A38m	191.50	21.30	0.0012	0.000	**0.000**
BN_AT_FuG_A20rv	190.02	16.62	0.0013	0.000	**0.002**
BN_AT_PhG_A35_36r	187.21	16.28	0.0013	0.000	**0.002**
BN_AT_PhG_A28_34	185.89	19.48	0.0016	0.000	**0.001**
BN_AT_PhG_TI	188.30	27.65	0.0016	0.000	**0.000**

Statistical summary of the age term from linear mixed models adjusted for sex and education. Trends are based on estimated marginal means for age-associated functional changes. Bolded *P*-values survived the Holm-Bonferroni correction ($P < 0.05$).
Abbreviations: ddf, approximate denominator degrees of freedom.

<u>Supplementary Table 10.</u> Age-related parahippocampal connectivity with subregions of the posterior-medial (PM) network.

	ddf	*F*	trend	*P*	adjusted *P*
BN_PM_PhG_TL	179,37	38,22	-0,0023	0,000	**0,000**
BN_PM_FuG_A20rv	179,77	32,19	-0,0021	0,000	**0,000**
BN_PM_PhG_TH	190,48	32,62	-0,0021	0,000	**0,000**
BN_PM_PCun_dmPOS	191,43	23,23	-0,0017	0,000	**0,000**
BN_PM_CG_A23v	184,53	23,75	-0,0015	0,000	**0,000**
BN_PM_OrG_A14m	183,14	21,88	-0,0013	0,000	**0,000**
BN_PM_PCun_A31	186,82	20,60	-0,0013	0,000	**0,000**
BN_PM_SFG_A8dl	182,70	16,24	-0,0012	0,000	**0,003**
BN_PM_Hipp_rHipp	178,89	17,59	-0,0012	0,000	**0,002**
BN_PM_MFG_A9_46d	182,44	13,04	-0,0011	0,000	**0,014**
BN_PM_IPL_A39rv	185,61	18,58	-0,0011	0,000	**0,001**
BN_PM_OrG_A11m	183,10	13,71	-0,0011	0,000	**0,010**
BN_PM_PhG_A28_34	174,13	12,44	-0,0011	0,001	**0,018**
BN_PM_MFG_A8vl	190,21	7,46	-0,0011	0,007	0,173
BN_PM_CG_A23c	171,68	19,99	-0,0010	0,000	**0,001**
BN_PM_CG_A23d	187,58	11,63	-0,0010	0,001	**0,025**
BN_PM_IPL_A39rd	182,06	6,01	-0,0010	0,015	0,281
BN_PM_PhG_A35_36c	176,14	12,60	-0,0010	0,000	**0,017**
BN_PM_OrG_A13	181,24	15,71	-0,0009	0,000	**0,004**
BN_PM_IPL_A39c	186,57	10,09	-0,0009	0,002	0,052
BN_PM_MVOcC_rLinG	184,83	6,83	-0,0008	0,010	0,224
BN_PM_PCun_A7m	182,24	6,58	-0,0008	0,011	0,245
BN_PM_SFG_A10m	185,00	9,71	-0,0007	0,002	0,062
BN_PM_MVOcC_vmPOS	190,96	6,28	-0,0007	0,013	0,274
BN_PM_MTG_A37dl	186,84	3,50	-0,0006	0,063	0,816
BN_PM_PCun_A5m	186,02	7,67	-0,0006	0,006	0,161
BN_PM_FuG_A37mv	184,19	4,45	-0,0006	0,036	0,545
BN_PM_Tha_cTtha	180,21	10,33	-0,0005	0,002	**0,048**
BN_PM_Tha_Otha	187,88	9,56	-0,0005	0,002	0,064
BN_PM_CG_A32sg	181,56	6,05	-0,0005	0,015	0,281
BN_PM_SPL_A7r	175,51	4,26	-0,0005	0,041	0,567
BN_PM_CG_A24rv	179,77	8,09	-0,0005	0,005	0,134
BN_PM_SFG_A9m	195,88	2,49	-0,0004	0,116	1,000
BN_PM_Hipp_cHipp	184,08	6,17	-0,0004	0,014	0,278
BN_PM_FuG_A37lv	184,81	2,35	-0,0004	0,127	1,000
BN_PM_LOcC_lsOccG	184,34	1,73	-0,0004	0,189	1,000
BN_PM_MFG_A6vl	179,31	0,94	-0,0004	0,334	1,000
BN_PM_Tha_PPtha	180,14	5,66	-0,0004	0,018	0,313
BN_PM_LOcC_msOccG	177,72	1,75	-0,0004	0,188	1,000
BN_PM_PCL_A1_2_3ll	180,66	0,51	-0,0001	0,477	1,000
BN_PM_SPL_A7c	180,20	0,12	-0,0001	0,730	1,000
BN_PM_MVOcC_rCunG	174,29	0,08	-0,0001	0,784	1,000
BN_PM_LOcC_V5_MT	185,83	0,21	0,0001	0,647	1,000
BN_PM_LOcC_mOccG	185,90	0,27	0,0001	0,604	1,000
BN_PM_LOcC_OPC	166,53	3,03	0,0003	0,084	0,921
BN_PM_MVOcC_cCunG	164,53	3,30	0,0004	0,071	0,855
BN_PM_MVOcC_cLinG	178,38	4,89	0,0005	0,028	0,453
BN_PM_LOcC_iOccG	179,76	7,20	0,0006	0,008	0,191

Statistical summary of the age term from linear mixed models adjusted for sex and education. Trends are based on estimated marginal means for age-associated functional changes. Bolded *P*-values survived the Holm-Bonferroni correction ($P < 0.05$).
Abbreviations: ddf, approximate denominator degrees of freedom.

<u>Supplementary Table 11</u>. Replication analyses of baseline differences between groups across the Alzheimer's continuum using different approaches to address potential power bias from the small sample of Aβ-positive cognitively unimpaired older adults. Statistical summary of functional connectivity in the anterior-temporal (AT) and posterior-medial (PM) networks across stages. Modelling methods: **(1)** Main text analyses distinguishing Aβ-negative from Aβ-positive cognitively unimpaired older adults; **(2)** Supplementary analysis merging Aβ-negative and Aβ-positive cognitively unimpaired older adults; **(3)** Supplementary analysis excluding Aβ-positive cognitively unimpaired older adults. *P*-values after adjustment for mean framewise displacement: $^{*}P < 0.05$; $^{t}0.05 < P < 0.1$

		Group effect		MCI - CU post hoc contrast		AD - CU post hoc contrast	
	Model	*F*	*P*	*t*	*P*	*t*	*P*
AT	1	3.3	**0.02**	2.5	**0.01***	2.3	**0.02**^{t}
	2	5.0	**0.009***	2.6	**0.01***	2.4	**0.02**^{t}
	3	4.3	**0.02**^{t}	2.4	**0.02***	2.3	**0.02**^{t}
PM	1	0.9	0.5				
	2	1.3	0.3				
	3	1.3	0.3				

Abbreviation: AD, Alzheimer's disease, CU, cognitively unimpaired, MCI, mild cognitive impairment.

<u>Supplementary Table 12</u>. Replication analyses of differences over time between groups across the Alzheimer's continuum using different approaches to address potential power bias from the small sample of Aβ-positive cognitively unimpaired older adults. Statistical summary of functional connectivity in the anterior-temporal (AT) and posterior-medial (PM) networks across stages. Modelling methods: **(1)** Main text analyses distinguishing Aβ-negative from Aβ-positive cognitively unimpaired older adults; **(2)** Supplementary analysis merging Aβ-negative and Aβ-positive cognitively unimpaired older adults; **(3)** Supplementary analysis excluding Aβ-positive cognitively unimpaired older adults.

		Group x time interaction	
	Model	*F*	*P*
AT	1	1.0	0.4
	2	0.6	0.5
	3	0.8	0.5
PM	1	1.5	0.2
	2	2.3	0.1
	3	2.3	0.1

Supplementary Table 13. Functional connectivity between the perirhinal cortex and subregions of the anterior-temporal (AT) network associated with cerebral amyloid uptake.

	ddf	*F*	trend	*P*	adjusted *P*
BN_AT_ITG_A20il	130.15	24.61	0.2455	0.000	**0.000**
BN_AT_ITG_A20iv	128.76	20.32	0.3025	0.000	**0.000**
BN_AT_ITG_A20r	127.62	16.99	0.1907	0.000	**0.001**
BN_AT_STG_TE10_TE12	200.00	16.61	0.1390	0.000	**0.001**
BN_AT_MTG_A21r	129.92	15.29	0.1847	0.000	**0.003**
BN_AT_Hipp_cHipp	133.03	13.22	0.2295	0.000	**0.007**
BN_AT_FuG_A20rv	131.96	12.34	0.2052	0.001	**0.011**
BN_AT_PhG_A28_34	135.20	11.27	0.2533	0.001	**0.017**
BN_AT_PhG_TL	127.52	9.87	0.1943	0.002	**0.033**
BN_AT_PhG_A35_36r	131.07	9.87	0.2131	0.002	**0.033**
BN_AT_Hipp_rHipp	133.17	8.84	0.1673	0.004	**0.049**
BN_AT_STG_A38l	131.15	7.61	0.1001	0.007	0.086
BN_AT_INS_vld_vlg	135.66	7.56	0.0851	0.007	0.086
BN_AT_PhG_TI	134.29	5.83	0.1555	0.017	0.188
BN_AT_STG_A38m	133.24	5.47	0.1147	0.021	0.209
BN_AT_Amyg_mAmyg	128.82	5.03	0.1134	0.027	0.239
BN_AT_PhG_A35_36c	129.79	4.76	0.1622	0.031	0.248
BN_AT_Amyg_lAmyg	130.88	4.59	0.1107	0.034	0.248
BN_AT_BG_NAC	200.00	1.64	0.0419	0.202	1.000
BN_AT_OrG_A12_47o	131.99	1.53	0.0485	0.218	1.000
BN_AT_ITG_A37elv	132.26	0.18	-0.0192	0.669	1.000
BN_AT_OrG_A11l	129.23	0.15	0.0150	0.695	1.000
BN_AT_ITG_A37vl	134.69	0.13	-0.0166	0.714	1.000
BN_AT_FuG_A37lv	127.41	0.01	-0.0042	0.911	1.000

Statistical summary of the amyloid term from linear mixed models adjusted for age, sex, education and follow time. Trends are based on estimated marginal means. Bolded *P*-values survived the Holm-Bonferroni correction ($P < 0.05$).
Abbreviations: ddf, approximate denominator degrees of freedom.

<u>Supplementary Table 14.</u> Functional connectivity between the perirhinal cortex and subregions of the anterior-temporal (AT) network associated with glucose metabolism.

	ddf	*F*	trend	*P*	adjusted *P*
BN_AT_ITG_A20il	126.67	31.42	-0.1429	0.000	**0.000**
BN_AT_Hipp_cHipp	132.05	29.30	-0.1663	0.000	**0.000**
BN_AT_MTG_A21r	125.62	28.14	-0.1212	0.000	**0.000**
BN_AT_Hipp_rHipp	130.00	22.07	-0.1291	0.000	**0.000**
BN_AT_STG_TE10_TE12	212.00	21.17	-0.0786	0.000	**0.000**
BN_AT_ITG_A20r	123.06	20.94	-0.1080	0.000	**0.000**
BN_AT_Amyg_lAmyg	125.71	18.09	-0.1061	0.000	**0.001**
BN_AT_PhG_A35_36r	128.64	18.08	-0.1439	0.000	**0.001**
BN_AT_ITG_A20iv	130.89	15.22	-0.1384	0.000	**0.002**
BN_AT_Amyg_mAmyg	128.84	14.32	-0.0949	0.000	**0.004**
BN_AT_PhG_A28_34	133.96	13.86	-0.1409	0.000	**0.004**
BN_AT_FuG_A20rv	131.37	13.83	-0.1116	0.000	**0.004**
BN_AT_INS_vId_vIg	129.94	13.74	-0.0574	0.000	**0.004**
BN_AT_PhG_A35_36c	130.65	12.91	-0.1335	0.000	**0.005**
BN_AT_STG_A38l	131.28	11.49	-0.0616	0.001	**0.009**
BN_AT_PhG_TI	133.64	10.05	-0.1048	0.002	**0.017**
BN_AT_PhG_TL	132.35	10.01	-0.0967	0.002	**0.017**
BN_AT_STG_A38m	131.58	8.47	-0.0736	0.004	**0.030**
BN_AT_BG_NAC	212.00	6.43	-0.0410	0.012	0.072
BN_AT_OrG_A12_47o	127.63	4.42	-0.0408	0.037	0.187
BN_AT_OrG_A11l	118.87	4.17	-0.0382	0.043	0.187
BN_AT_FuG_A37lv	124.08	1.61	-0.0242	0.207	0.620
BN_AT_ITG_A37elv	131.65	0.37	-0.0141	0.545	1.000
BN_AT_ITG_A37vl	127.79	0.02	-0.0034	0.884	1.000

Statistical summary of the metabolism term from linear mixed models adjusted for age, sex, education and follow time. Trends are based on estimated marginal means. Bolded *P*-values survived the Holm-Bonferroni correction (*P* < 0.05).
Abbreviations: ddf, approximate denominator degrees of freedom.

<u>Supplementary Table 15.</u> Functional connectivity between the perirhinal cortex and subregions of the anterior-temporal (AT) network associated with hippocampal volume.

	ddf	*F*	trend	*P*	adjusted *P*
BN_AT_Hipp_cHipp	113.39	21.92	-0.1473	0.000	**0.000**
BN_AT_Amyg_lAmyg	112.80	13.78	-0.0916	0.000	**0.007**
BN_AT_Amyg_mAmyg	107.80	11.36	-0.0827	0.001	**0.023**
BN_AT_PhG_A28_34	118.28	10.67	-0.1271	0.001	**0.030**
BN_AT_Hipp_rHipp	113.86	10.20	-0.0922	0.002	**0.036**
BN_AT_STG_A38m	119.52	9.63	-0.0779	0.002	**0.045**
BN_AT_ITG_A20r	109.43	9.53	-0.0753	0.003	**0.046**
BN_AT_ITG_A20il	112.32	9.25	-0.0841	0.003	**0.050**
BN_AT_MTG_A21r	109.51	7.23	-0.0654	0.008	0.133
BN_AT_PhG_A35_36r	112.35	6.86	-0.0923	0.010	0.150
BN_AT_PhG_TL	113.18	6.78	-0.0819	0.010	0.150
BN_AT_STG_TE10_TE12	104.84	6.53	-0.0422	0.012	0.157
BN_AT_PhG_TI	119.84	6.14	-0.0819	0.015	0.175
BN_AT_STG_A38l	116.32	5.36	-0.0434	0.022	0.246
BN_AT_FuG_A20rv	113.84	5.13	-0.0700	0.025	0.253
BN_AT_PhG_A35_36c	114.31	4.45	-0.0822	0.037	0.334
BN_AT_ITG_A20iv	112.18	3.68	-0.0710	0.057	0.460
BN_AT_OrG_A11l	115.91	2.13	0.0288	0.147	1.000
BN_AT_INS_vId_vIg	116.88	1.81	-0.0211	0.181	1.000
BN_AT_BG_NAC	103.34	1.25	-0.0178	0.266	1.000
BN_AT_OrG_A12_47o	122.12	0.63	0.0168	0.431	1.000
BN_AT_ITG_A37vl	116.40	0.31	0.0126	0.579	1.000
BN_AT_ITG_A37elv	117.39	0.26	0.0116	0.613	1.000
BN_AT_FuG_A37lv	109.80	0.03	-0.0030	0.872	1.000

Statistical summary of the hippocampal volume term from linear mixed models adjusted for age, sex, education and follow time. Trends are based on estimated marginal means. Bolded *P*-values survived the Holm-Bonferroni correction ($P < 0.05$).
Abbreviations: ddf, approximate denominator degrees of freedom.

<u>Supplementary Table 16.</u> Functional connectivity between the perirhinal cortex and subregions of the anterior-temporal (AT) network associated with Mini-Mental Scale Examination (MMSE) scores.

	ddf	*F*	trend	*P*	adjusted *P*
BN_AT_ITG_A20il	164.49	28.08	-0.0086	0.000	**0.000**
BN_AT_MTG_A21r	159.78	23.11	-0.0071	0.000	**0.000**
BN_AT_STG_TE10_TE12	231.00	16.80	-0.0046	0.000	**0.001**
BN_AT_Hipp_cHipp	171.10	12.97	-0.0072	0.000	**0.009**
BN_AT_INS_vId_vIg	160.82	12.37	-0.0035	0.001	**0.011**
BN_AT_ITG_A20r	158.66	11.62	-0.0053	0.001	**0.016**
BN_AT_STG_A38l	161.00	11.01	-0.0039	0.001	**0.020**
BN_AT_ITG_A20iv	163.44	8.80	-0.0068	0.003	0.059
BN_AT_Hipp_rHipp	167.77	8.35	-0.0053	0.004	0.070
BN_AT_PhG_TL	167.26	7.70	-0.0054	0.006	0.092
BN_AT_Amyg_lAmyg	162.07	7.24	-0.0044	0.008	0.110
BN_AT_PhG_A35_36r	165.00	7.00	-0.0058	0.009	0.116
BN_AT_PhG_A28_34	171.31	5.25	-0.0056	0.023	0.278
BN_AT_PhG_A35_36c	166.57	5.19	-0.0055	0.024	0.278
BN_AT_FuG_A20rv	166.23	5.14	-0.0044	0.025	0.278
BN_AT_Amyg_mAmyg	161.57	4.72	-0.0035	0.031	0.281
BN_AT_PhG_TI	167.47	4.67	-0.0045	0.032	0.281
BN_AT_BG_NAC	150.78	3.68	-0.0020	0.057	0.398
BN_AT_FuG_A37lv	155.68	2.72	-0.0020	0.101	0.607
BN_AT_STG_A38m	166.26	1.99	-0.0024	0.161	0.804
BN_AT_OrG_A12_47o	164.47	1.11	-0.0014	0.294	1.000
BN_AT_ITG_A37vl	161.64	0.89	-0.0014	0.347	1.000
BN_AT_ITG_A37elv	162.16	0.67	-0.0012	0.413	1.000
BN_AT_OrG_A11l	158.82	0.01	-0.0001	0.914	1.000

Statistical summary of the MMSE term from linear mixed models adjusted for age, sex, education and follow time. Trends are based on estimated marginal means. Bolded *P*-values survived the Holm-Bonferroni correction ($P < 0.05$).
Abbreviations: ddf, approximate denominator degrees of freedom.

<u>Supplementary Table 17.</u> Functional connectivity between the perirhinal cortex and subregions of the anterior-temporal (AT) network associated with Mattis Dementia Rating Scale (MDRS) scores.

	ddf	*F*	trend	*P*	adjusted *P*
BN_AT_ITG_A20il	162.93	27.79	-0.0031	0.000	**0.000**
BN_AT_STG_TE10_TE12	160.11	17.11	-0.0018	0.000	**0.001**
BN_AT_INS_vId_vIg	161.89	16.81	-0.0016	0.000	**0.001**
BN_AT_MTG_A21r	159.00	15.90	-0.0022	0.000	**0.002**
BN_AT_Hipp_cHipp	171.43	13.14	-0.0026	0.000	**0.008**
BN_AT_ITG_A20r	159.28	12.69	-0.0020	0.000	**0.009**
BN_AT_Hipp_rHipp	169.42	11.88	-0.0023	0.001	**0.013**
BN_AT_PhG_A35_36r	166.62	9.92	-0.0025	0.002	**0.033**
BN_AT_PhG_A28_34	171.82	9.16	-0.0027	0.003	**0.046**
BN_AT_Amyg_lAmyg	164.36	8.09	-0.0017	0.005	0.075
BN_AT_BG_NAC	158.04	8.08	-0.0011	0.005	0.075
BN_AT_PhG_TL	169.49	6.92	-0.0019	0.009	0.121
BN_AT_ITG_A20iv	160.26	6.59	-0.0022	0.011	0.134
BN_AT_FuG_A20rv	166.83	6.43	-0.0018	0.012	0.134
BN_AT_STG_A38l	158.51	6.22	-0.0011	0.014	0.136
BN_AT_PhG_TI	169.08	6.00	-0.0019	0.015	0.138
BN_AT_Amyg_mAmyg	165.22	5.50	-0.0014	0.020	0.162
BN_AT_PhG_A35_36c	169.56	5.38	-0.0021	0.022	0.162
BN_AT_FuG_A37lv	158.82	2.39	-0.0007	0.124	0.746
BN_AT_OrG_A12_47o	168.22	1.90	-0.0007	0.170	0.850
BN_AT_STG_A38m	168.02	1.73	-0.0008	0.190	0.850
BN_AT_ITG_A37vl	166.52	1.11	-0.0006	0.295	0.884
BN_AT_ITG_A37elv	166.78	1.02	-0.0006	0.314	0.884
BN_AT_OrG_A11l	162.71	0.51	-0.0004	0.475	0.884

Statistical summary of the MDRS term from linear mixed models adjusted for age, sex, education and follow time. Trends are based on estimated marginal means. Bolded *P*-values survived the Holm-Bonferroni correction ($P < 0.05$).
Abbreviations: ddf, approximate denominator degrees of freedom.

<u>Supplementary Table 18.</u> Functional connectivity between the parahippocampal cortex and subregions of posterior-medial (PM) network associated with cerebral amyloid uptake.

	ddf	*F*	trend	*P*	adjusted *P*
BN_PM_PhG_A35_36c	134.23	20.04	0.2364	0.000	**0.001**
BN_PM_FuG_A37mv	138.89	18.47	0.2137	0.000	**0.001**
BN_PM_MVOcC_rLinG	133.48	15.13	0.2036	0.000	**0.006**
BN_PM_Tha_Otha	200.00	11.72	0.1556	0.001	**0.029**
BN_PM_LOcC_iOccG	129.60	11.67	0.1723	0.001	**0.031**
BN_PM_FuG_A37lv	132.15	10.96	0.1439	0.001	**0.043**
BN_PM_SFG_A8dl	124.55	10.63	-0.1161	0.001	0.050
BN_PM_PhG_TH	134.90	9.34	0.1769	0.003	0.092
BN_PM_IPL_A39rd	120.49	7.69	-0.1420	0.006	0.212
BN_PM_SPL_A7c	133.80	7.41	-0.1125	0.007	0.235
BN_PM_LOcC_mOccG	125.04	6.42	0.1236	0.013	0.388
BN_PM_MFG_A8vl	125.18	6.40	-0.1086	0.013	0.388
BN_PM_MVOcC_cLinG	125.87	5.81	0.1207	0.017	0.505
BN_PM_FuG_A20rv	136.13	5.68	0.1372	0.019	0.521
BN_PM_Hipp_cHipp	133.19	4.20	0.0950	0.042	1.000
BN_PM_MFG_A9_46d	130.76	3.17	-0.0819	0.077	1.000
BN_PM_IPL_A39rv	112.62	2.77	-0.0712	0.099	1.000
BN_PM_LOcC_OPC	133.69	2.72	0.0874	0.101	1.000
BN_PM_PhG_TL	130.49	2.43	0.0871	0.121	1.000
BN_PM_MFG_A6vl	128.77	2.26	-0.0625	0.135	1.000
BN_PM_MVOcC_cCunG	129.00	2.09	0.0760	0.151	1.000
BN_PM_Hipp_rHipp	135.93	1.82	0.0851	0.179	1.000
BN_PM_PCL_A1_2_3ll	125.28	1.43	0.0418	0.233	1.000
BN_PM_IPL_A39c	129.61	1.33	-0.0469	0.250	1.000
BN_PM_ITG_A37vl	130.56	1.19	0.0635	0.278	1.000
BN_PM_LOcC_V5_MT	132.13	1.18	0.0500	0.280	1.000
BN_PM_CG_A32sg	127.64	1.05	-0.0489	0.306	1.000
BN_PM_OrG_A14m	131.88	1.00	-0.0447	0.320	1.000
BN_PM_PCun_A31	120.65	0.91	-0.0384	0.342	1.000
BN_PM_PCun_dmPOS	133.23	0.77	-0.0469	0.382	1.000
BN_PM_MVOcC_rCunG	132.12	0.73	0.0364	0.393	1.000
BN_PM_PCun_A7m	134.58	0.57	-0.0315	0.450	1.000
BN_PM_SPL_A7r	129.51	0.49	0.0256	0.485	1.000
BN_PM_CG_A23v	131.20	0.48	-0.0359	0.488	1.000
BN_PM_CG_A23d	126.57	0.33	-0.0247	0.565	1.000
BN_PM_LOcC_lsOccG	129.19	0.30	-0.0230	0.588	1.000
BN_PM_PCun_A5m	132.03	0.07	-0.0077	0.794	1.000
BN_PM_OrG_A11m	129.01	0.04	0.0092	0.844	1.000
BN_PM_MVOcC_vmPOS	132.75	0.03	0.0072	0.865	1.000
BN_PM_PCL_A4ll	200.00	0.00	0.0015	0.960	1.000
BN_PM_LOcC_msOccG	127.25	0.00	0.0006	0.989	1.000

Statistical summary of the amyloid term from linear mixed models adjusted for age, sex, education and follow time. Trends are based on estimated marginal means. Bolded *P*-values survived the Holm-Bonferroni correction ($P < 0.05$).
Abbreviations: ddf, approximate denominator degrees of freedom.

<u>Supplementary Table 19.</u> Functional connectivity between the parahippocampal cortex and subregions of posterior-medial (PM) network associated with glucose metabolism.

	ddf	*F*	trend	*P*	adjusted *P*
BN_PM_MVOcC_rLinG	126.98	17.31	-0.1070	0.000	**0.002**
BN_PM_FuG_A37mv	130.95	13.31	-0.0914	0.000	**0.015**
BN_PM_FuG_A37lv	124.01	12.24	-0.0752	0.001	**0.025**
BN_PM_Tha_Otha	117.73	11.58	-0.0776	0.001	**0.035**
BN_PM_LOcC_mOccG	119.74	11.38	-0.0822	0.001	**0.037**
BN_PM_LOcC_iOccG	124.90	10.38	-0.0834	0.002	0.058
BN_PM_LOcC_V5_MT	127.36	9.39	-0.0707	0.003	0.093
BN_PM_PhG_A35_36c	135.92	9.25	-0.0854	0.003	0.096
BN_PM_MVOcC_cLinG	119.94	9.13	-0.0751	0.003	0.101
BN_PM_FuG_A20rv	130.70	9.06	-0.0867	0.003	0.101
BN_PM_PhG_TL	128.11	8.29	-0.0793	0.005	0.145
BN_PM_PhG_TH	134.74	8.24	-0.0841	0.005	0.145
BN_PM_SFG_A8dl	122.41	7.72	0.0539	0.006	0.183
BN_PM_MFG_A8vl	121.70	5.73	0.0512	0.018	0.510
BN_PM_Hipp_cHipp	130.69	4.57	-0.0505	0.034	0.928
BN_PM_LOcC_OPC	126.21	3.93	-0.0529	0.050	1.000
BN_PM_IPL_A39rd	125.45	3.45	0.0532	0.066	1.000
BN_PM_PCL_A1_2_3ll	123.12	3.17	-0.0318	0.078	1.000
BN_PM_SPL_A7c	137.20	2.06	0.0324	0.153	1.000
BN_PM_MFG_A6vl	125.56	1.69	0.0282	0.196	1.000
BN_PM_MVOcC_cCunG	119.97	1.52	-0.0324	0.221	1.000
BN_PM_PCL_A4ll	212.00	1.42	-0.0180	0.235	1.000
BN_PM_CG_A23d	127.75	1.42	0.0264	0.236	1.000
BN_PM_MVOcC_rCunG	122.39	1.25	-0.0234	0.266	1.000
BN_PM_Hipp_rHipp	135.04	1.23	-0.0356	0.269	1.000
BN_PM_MFG_A9_46d	128.51	1.02	0.0242	0.314	1.000
BN_PM_PCun_A7m	130.08	0.75	0.0187	0.387	1.000
BN_PM_ITG_A37vl	124.45	0.65	-0.0229	0.423	1.000
BN_PM_LOcC_msOccG	128.60	0.56	-0.0185	0.457	1.000
BN_PM_OrG_A14m	133.81	0.25	0.0119	0.619	1.000
BN_PM_CG_A32sg	128.43	0.23	0.0122	0.635	1.000
BN_PM_PCun_A5m	128.30	0.14	-0.0057	0.708	1.000
BN_PM_CG_A23v	127.65	0.13	-0.0091	0.724	1.000
BN_PM_SPL_A7r	131.11	0.11	0.0066	0.736	1.000
BN_PM_LOcC_lsOccG	134.20	0.11	-0.0072	0.742	1.000
BN_PM_OrG_A11m	135.29	0.08	-0.0071	0.781	1.000
BN_PM_IPL_A39rv	116.68	0.07	0.0061	0.786	1.000
BN_PM_IPL_A39c	128.09	0.07	-0.0055	0.794	1.000
BN_PM_MVOcC_vmPOS	125.89	0.03	-0.0038	0.859	1.000
BN_PM_PCun_A31	122.91	0.03	0.0035	0.870	1.000
BN_PM_PCun_dmPOS	132.11	0.00	0.0013	0.961	1.000

Statistical summary of the metabolism term from linear mixed models adjusted for age, sex, education and follow time. Trends are based on estimated marginal means. Bolded *P*-values survived the Holm-Bonferroni correction ($P < 0.05$).
Abbreviations: ddf, approximate denominator degrees of freedom.

Supplementary Table 20. Functional connectivity between the parahippocampal cortex and subregions of posterior-medial (PM) network associated with hippocampal volume.

	ddf	*F*	trend	*P*	adjusted *P*
BN_PM_LOcC_iOccG	115.80	7.73	-0.0707	0.006	0.260
BN_PM_PhG_TL	113.94	5.77	-0.0669	0.018	0.698
BN_PM_MVOcC_rLinG	116.32	5.98	-0.0665	0.016	0.640
BN_PM_LOcC_mOccG	114.41	4.66	-0.0554	0.033	1.000
BN_PM_MVOcC_cLinG	110.68	4.77	-0.0534	0.031	1.000
BN_PM_FuG_A37mv	121.28	3.97	-0.0524	0.048	1.000
BN_PM_Tha_Otha	114.40	4.54	-0.0505	0.035	1.000
BN_PM_PhG_A35_36c	119.70	2.96	-0.0486	0.088	1.000
BN_PM_PhG_TH	119.38	1.45	-0.0368	0.230	1.000
BN_PM_LOcC_OPC	114.82	1.97	-0.0368	0.164	1.000
BN_PM_Hipp_rHipp	119.40	1.12	-0.0336	0.292	1.000
BN_PM_Hipp_cHipp	115.70	2.01	-0.0333	0.159	1.000
BN_PM_FuG_A20rv	118.69	1.26	-0.0330	0.264	1.000
BN_PM_LOcC_V5_MT	118.58	1.67	-0.0305	0.199	1.000
BN_PM_MVOcC_cCunG	110.29	1.35	-0.0294	0.248	1.000
BN_PM_PCL_A1_2_3ll	113.48	2.59	-0.0288	0.110	1.000
BN_PM_FuG_A37lv	114.67	0.86	-0.0206	0.357	1.000
BN_PM_LOcC_msOccG	113.27	0.30	-0.0139	0.584	1.000
BN_PM_PCL_A4ll	122.22	0.46	-0.0098	0.501	1.000
BN_PM_ITG_A37vl	106.13	0.08	-0.0081	0.778	1.000
BN_PM_LOcC_lsOccG	117.62	0.03	-0.0036	0.868	1.000
BN_PM_OrG_A11m	119.69	0.01	-0.0021	0.933	1.000
BN_PM_SPL_A7r	116.21	0.00	0.0014	0.945	1.000
BN_PM_MVOcC_rCunG	115.66	0.01	0.0022	0.918	1.000
BN_PM_OrG_A14m	122.05	0.05	0.0051	0.830	1.000
BN_PM_PCun_A5m	118.06	0.13	0.0055	0.715	1.000
BN_PM_CG_A32sg	116.13	0.12	0.0086	0.734	1.000
BN_PM_MFG_A6vl	111.50	0.25	0.0111	0.620	1.000
BN_PM_CG_A23d	113.10	0.49	0.0156	0.486	1.000
BN_PM_MVOcC_vmPOS	122.27	0.53	0.0158	0.468	1.000
BN_PM_PCun_dmPOS	118.69	0.41	0.0175	0.522	1.000
BN_PM_IPL_A39c	116.49	0.74	0.0184	0.391	1.000
BN_PM_MFG_A8vl	116.18	1.02	0.0223	0.315	1.000
BN_PM_PCun_A31	111.04	1.44	0.0251	0.232	1.000
BN_PM_CG_A23v	120.35	1.12	0.0273	0.293	1.000
BN_PM_MFG_A9_46d	119.88	1.43	0.0294	0.234	1.000
BN_PM_IPL_A39rv	107.48	2.14	0.0325	0.146	1.000
BN_PM_IPL_A39rd	115.51	1.36	0.0329	0.245	1.000
BN_PM_PCun_A7m	117.58	3.11	0.0375	0.080	1.000
BN_PM_SFG_A8dl	116.79	3.85	0.0391	0.052	1.000
BN_PM_SPL_A7c	121.76	3.96	0.0448	0.049	1.000

Statistical summary of the hippocampal volume term from linear mixed models adjusted for age, sex, education and follow time. Trends are based on estimated marginal means. Bolded *P*-values survived the Holm-Bonferroni correction ($P < 0.05$).
Abbreviations: ddf, approximate denominator degrees of freedom.

Supplementary Table 21. Functional connectivity between the parahippocampal cortex and subregions of posterior-medial (PM) network associated with Mini-Mental Scale Examination (MMSE) scores.

	ddf	*F*	trend	*P*	adjusted *P*
BN_PM_FuG_A37mv	163.71	18.94	-0.0071	0.000	**0.001**
BN_PM_MVOcC_rLinG	158.80	14.06	-0.0064	0.000	**0.009**
BN_PM_LOcC_iOccG	158.41	14.53	-0.0062	0.000	**0.008**
BN_PM_PhG_A35_36c	162.54	11.31	-0.0059	0.001	**0.036**
BN_PM_FuG_A20rv	162.90	9.65	-0.0057	0.002	0.076
BN_PM_FuG_A37lv	158.19	16.46	-0.0056	0.000	**0.003**
BN_PM_Tha_Otha	160.87	11.31	-0.0052	0.001	**0.036**
BN_PM_MVOcC_cLinG	155.37	9.76	-0.0050	0.002	0.074
BN_PM_PhG_TH	165.32	5.98	-0.0046	0.016	0.451
BN_PM_LOcC_mOccG	157.07	7.97	-0.0045	0.005	0.172
BN_PM_LOcC_OPC	158.31	6.81	-0.0044	0.010	0.297
BN_PM_PhG_TL	159.37	5.37	-0.0041	0.022	0.589
BN_PM_Hipp_cHipp	160.63	7.04	-0.0040	0.009	0.272
BN_PM_Hipp_rHipp	164.75	3.29	-0.0036	0.071	1.000
BN_PM_LOcC_V5_MT	161.16	3.97	-0.0030	0.048	1.000
BN_PM_MVOcC_cCunG	154.04	2.78	-0.0028	0.098	1.000
BN_PM_ITG_A37vl	153.48	2.02	-0.0026	0.157	1.000
BN_PM_PCL_A1_2_3ll	157.06	4.01	-0.0024	0.047	1.000
BN_PM_OrG_A11m	166.93	0.42	-0.0010	0.519	1.000
BN_PM_MVOcC_rCunG	159.34	0.51	-0.0010	0.474	1.000
BN_PM_PCL_A4ll	167.72	0.59	-0.0008	0.444	1.000
BN_PM_CG_A23v	163.45	0.03	-0.0003	0.867	1.000
BN_PM_PCun_A31	158.37	0.01	-0.0001	0.941	1.000
BN_PM_CG_A32sg	163.39	0.02	0.0002	0.888	1.000
BN_PM_LOcC_lsOccG	172.49	0.03	0.0002	0.856	1.000
BN_PM_LOcC_msOccG	165.95	0.03	0.0003	0.864	1.000
BN_PM_OrG_A14m	169.94	0.05	0.0003	0.817	1.000
BN_PM_PCun_A5m	161.35	0.20	0.0004	0.657	1.000
BN_PM_IPL_A39c	161.69	0.19	0.0006	0.661	1.000
BN_PM_MVOcC_vmPOS	165.52	0.26	0.0007	0.612	1.000
BN_PM_SPL_A7r	160.57	0.57	0.0009	0.451	1.000
BN_PM_CG_A23d	158.27	0.49	0.0010	0.486	1.000
BN_PM_IPL_A39rv	153.63	0.96	0.0014	0.330	1.000
BN_PM_PCun_dmPOS	163.50	1.04	0.0018	0.308	1.000
BN_PM_MFG_A6vl	158.11	1.93	0.0019	0.166	1.000
BN_PM_MFG_A9_46d	164.11	1.55	0.0020	0.215	1.000
BN_PM_PCun_A7m	160.56	2.84	0.0023	0.094	1.000
BN_PM_MFG_A8vl	160.31	4.04	0.0029	0.046	1.000
BN_PM_SPL_A7c	166.27	5.50	0.0034	0.020	0.565
BN_PM_IPL_A39rd	160.78	3.93	0.0036	0.049	1.000
BN_PM_SFG_A8dl	160.47	9.20	0.0039	0.003	0.093

Statistical summary of the MMSE term from linear mixed models adjusted for age, sex, education and follow time. Trends are based on estimated marginal means. Bolded *P*-values survived the Holm-Bonferroni correction ($P < 0.05$).
Abbreviations: ddf, approximate denominator degrees of freedom.

Supplementary Table 22. Functional connectivity between the parahippocampal cortex and subregions of posterior-medial (PM) network associated with Mattis Dementia Rating Scale (MDRS) scores.

	ddf	*F*	trend	*P*	adjusted *P*
BN_PM_PhG_A35_36c	158.56	9.62	-0.0021	0.002	0.089
BN_PM_FuG_A37mv	166.43	10.36	-0.0020	0.002	0.064
BN_PM_MVOcC_rLinG	162.86	9.17	-0.0020	0.003	0.100
BN_PM_LOcC_iOccG	157.78	9.49	-0.0019	0.002	0.090
BN_PM_Tha_Otha	155.58	8.91	-0.0018	0.003	0.112
BN_PM_PhG_TH	164.05	5.96	-0.0017	0.016	0.504
BN_PM_FuG_A37lv	162.23	10.26	-0.0017	0.002	0.066
BN_PM_MVOcC_cLinG	155.78	7.64	-0.0017	0.006	0.211
BN_PM_Hipp_cHipp	158.24	5.81	-0.0014	0.017	0.530
BN_PM_LOcC_OPC	156.52	4.49	-0.0014	0.036	1.000
BN_PM_FuG_A20rv	163.02	3.63	-0.0014	0.058	1.000
BN_PM_MVOcC_cCunG	152.40	3.22	-0.0011	0.075	1.000
BN_PM_LOcC_mOccG	158.91	3.12	-0.0011	0.079	1.000
BN_PM_PhG_TL	160.22	2.20	-0.0010	0.140	1.000
BN_PM_ITG_A37vl	157.01	1.24	-0.0008	0.267	1.000
BN_PM_Hipp_rHipp	164.89	0.97	-0.0008	0.325	1.000
BN_PM_LOcC_V5_MT	160.97	1.06	-0.0006	0.304	1.000
BN_PM_PCL_A1_2_3ll	162.93	1.54	-0.0005	0.216	1.000
BN_PM_OrG_A11m	167.88	0.26	-0.0003	0.611	1.000
BN_PM_MVOcC_rCunG	160.00	0.33	-0.0003	0.567	1.000
BN_PM_PCL_A4ll	173.39	0.00	0.0000	0.989	1.000
BN_PM_CG_A23v	165.72	0.00	0.0000	0.963	1.000
BN_PM_CG_A32sg	164.88	0.04	0.0001	0.845	1.000
BN_PM_PCun_A31	162.19	0.22	0.0002	0.643	1.000
BN_PM_LOcC_lsOccG	170.57	0.37	0.0003	0.543	1.000
BN_PM_PCun_A5m	167.05	1.06	0.0004	0.306	1.000
BN_PM_LOcC_msOccG	163.58	0.52	0.0004	0.474	1.000
BN_PM_OrG_A14m	170.72	0.54	0.0004	0.465	1.000
BN_PM_CG_A23d	163.18	0.88	0.0005	0.348	1.000
BN_PM_IPL_A39c	161.79	1.07	0.0005	0.303	1.000
BN_PM_MVOcC_vmPOS	161.25	1.06	0.0006	0.304	1.000
BN_PM_SPL_A7r	162.31	1.53	0.0006	0.218	1.000
BN_PM_MFG_A6vl	162.50	2.36	0.0008	0.126	1.000
BN_PM_IPL_A39rv	158.52	2.36	0.0008	0.126	1.000
BN_PM_MFG_A9_46d	168.23	2.17	0.0009	0.142	1.000
BN_PM_PCun_A7m	164.66	2.83	0.0009	0.095	1.000
BN_PM_PCun_dmPOS	164.64	2.08	0.0009	0.151	1.000
BN_PM_MFG_A8vl	164.78	5.33	0.0012	0.022	0.666
BN_PM_IPL_A39rd	163.89	3.34	0.0013	0.070	1.000
BN_PM_SFG_A8dl	165.21	9.59	0.0015	0.002	0.089
BN_PM_SPL_A7c	168.36	9.32	0.0016	0.003	0.095

Statistical summary of the MDRS term from linear mixed models adjusted for age, sex, education and follow time. Trends are based on estimated marginal means. Bolded *P*-values survived the Holm-Bonferroni correction ($P < 0.05$).
Abbreviations: ddf, approximate denominator degrees of freedom.

Supplementary Figures

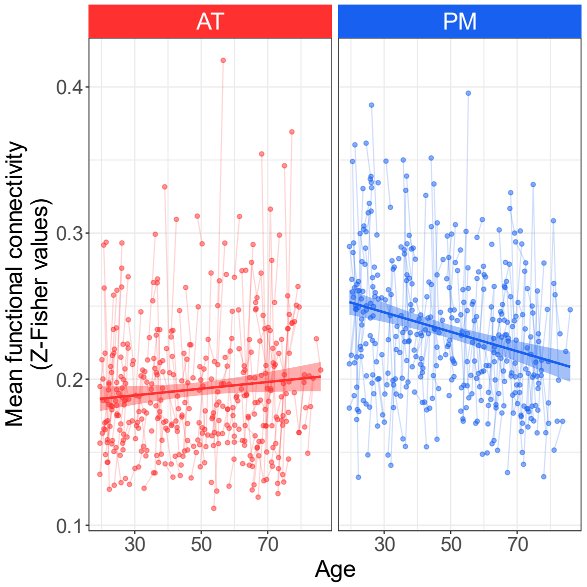

Supplementary Figure 1. Age-related trajectories of functional connectivity in the anterior-temporal (AT) and posterior-medial (PM) networks across the adult lifespan.
Regression lines were added to aid visualization, but they do not account for interdependencies within participants and covariates, unlike the model predictions plotted in the main text.

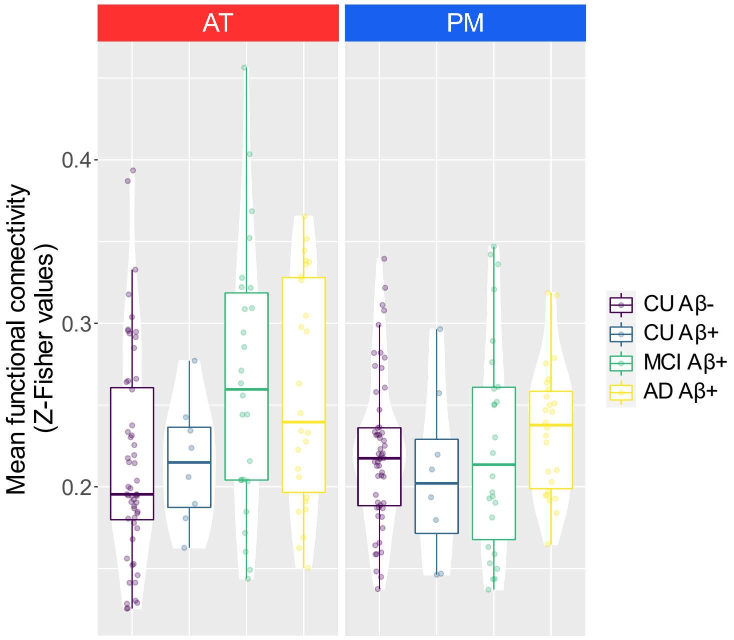

Supplementary Figure 2. Baseline group differences in functional connectivity within the anterior-temporal (AT) and posterior-medial (PM) networks across the Alzheimer's continuum.
Boxplot estimates do not account for covariates, unlike the model predictions plotted in the main text.
Abbreviations: AD, Alzheimer's disease; CU, cognitively unimpaired; MCI, mild cognitive impairment.

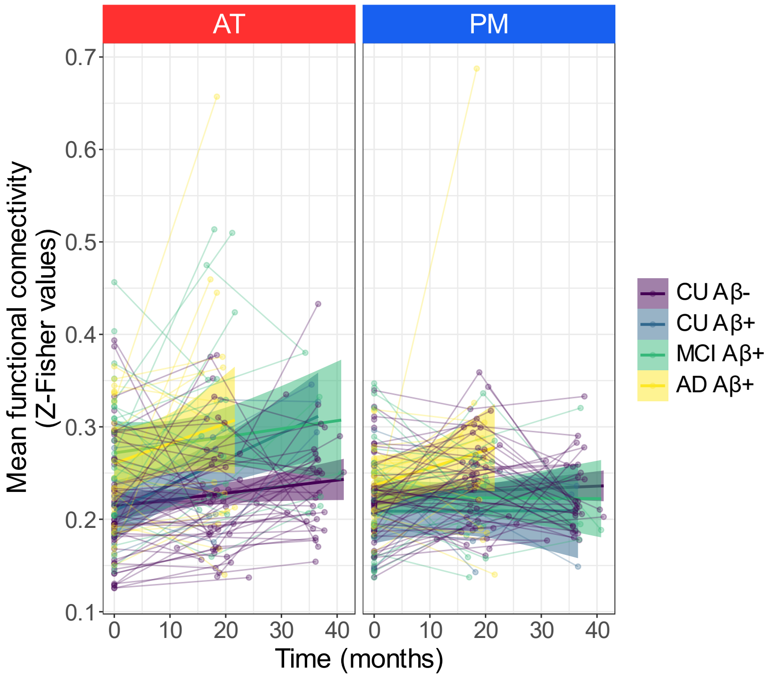

Supplementary Figure 2. Group differences in functional connectivity changes over time within the anterior-temporal (AT) and posterior-medial (PM) networks across the Alzheimer's continuum.
Regression lines were added to aid visualization, but they do not account for interdependencies within participants and covariates, unlike the model predictions plotted in the main text.
Abbreviations: AD, Alzheimer's disease; CU, cognitively unimpaired; MCI, mild cognitive impairment.

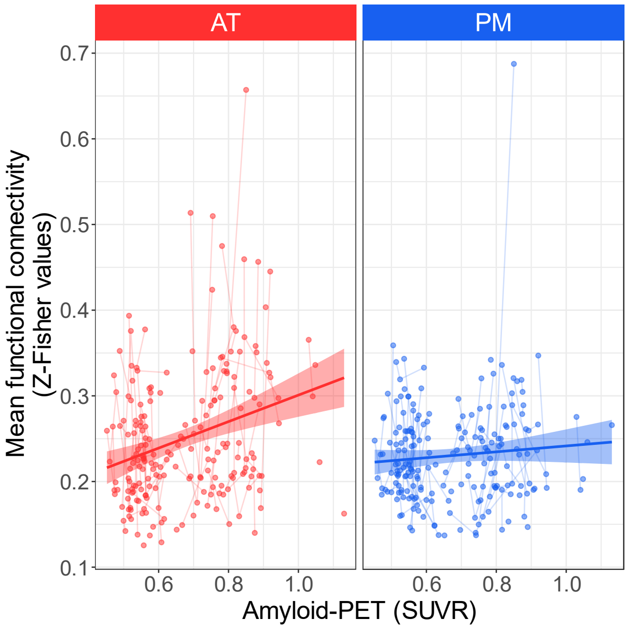

Supplementary Figure 3. Association between cerebral amyloid uptake and functional connectivity in the anterior-temporal (AT) and posterior-medial (PM) networks among older adults, from healthy to Alzheimer's demented.
Regression lines were added to aid visualization, but they do not account for interdependencies within participants and covariates, unlike the model predictions plotted in the main text.
Abbreviations: SUVR, standardized uptake value ratio.

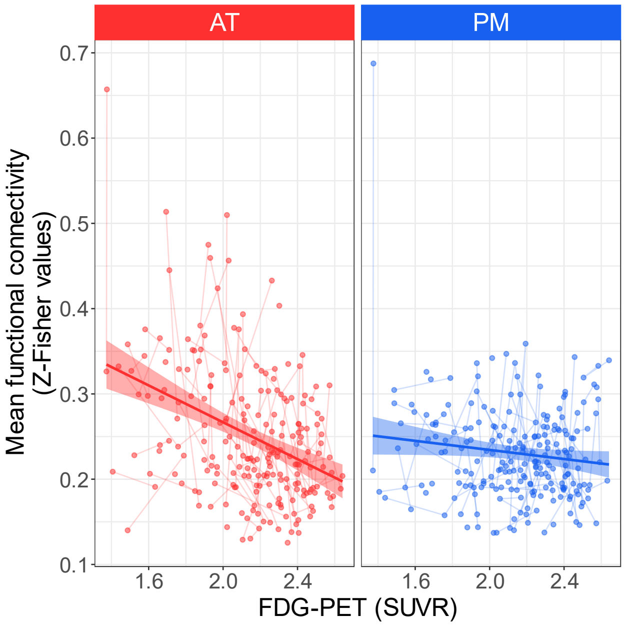

<u>Supplementary Figure 5</u>. Association between cerebral glucose metabolism and functional connectivity in the anterior-temporal (AT) and posterior-medial (PM) networks among older adults, from healthy to Alzheimer's demented.
Regression lines were added to aid visualization, but they do not account for interdependencies within participants and covariates, unlike the model predictions plotted in the main text.
Abbreviations: SUVR, standardized uptake value ratio.

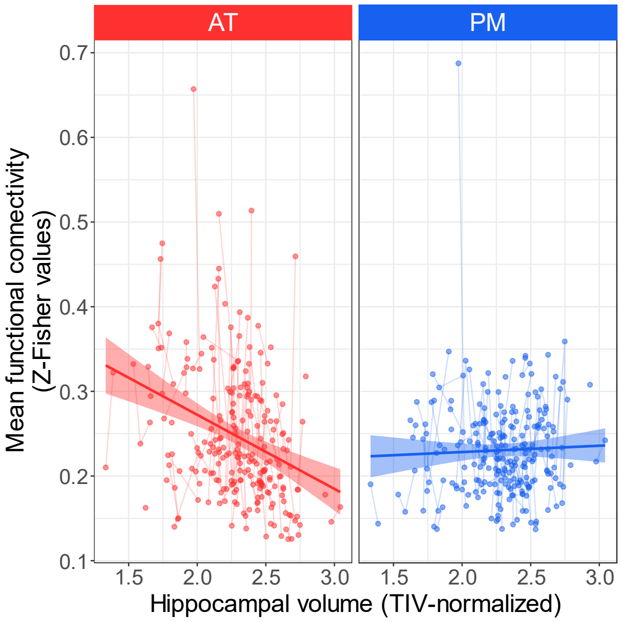

<u>Supplementary Figure 6</u>. Association between hippocampal volume and functional connectivity in the anterior-temporal (AT) and posterior-medial (PM) networks among older adults, from healthy to Alzheimer's demented.
Regression lines were added to aid visualization, but they do not account for interdependencies within participants and covariates, unlike the model predictions plotted in the main text.
Abbreviations: TIV, total intracranial volume.

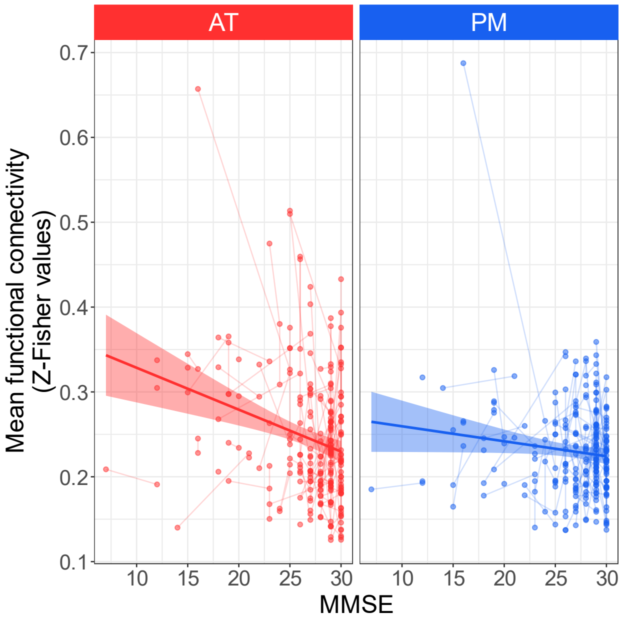

Supplementary Figure 7. Association between mini-mental state examination scores and functional connectivity in the anterior-temporal (AT) and posterior-medial (PM) networks among older adults, from healthy to Alzheimer's demented. Regression lines were added to aid visualization, but they do not account for interdependencies within participants and covariates, unlike the model predictions plotted in the main text.
Abbreviations: MMSE, Mini-Mental State Examination.

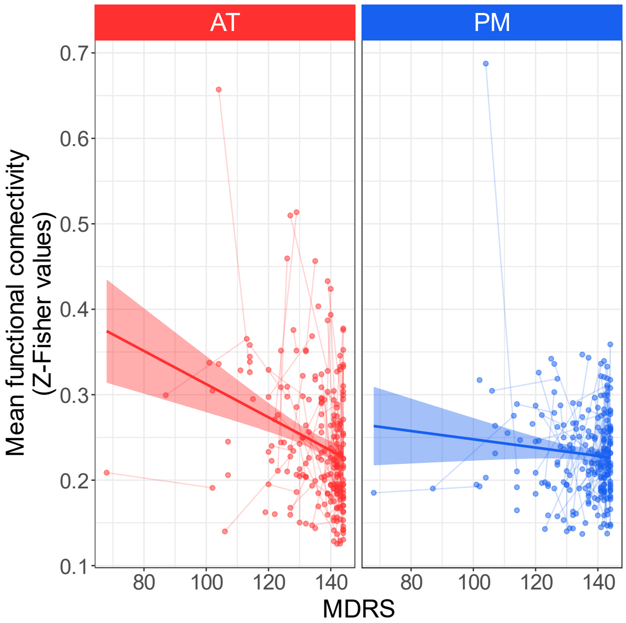

Supplementary Figure 8. Association between Mattis dementia rating scale scores and functional connectivity in the anterior-temporal (AT) and posterior-medial (PM) networks among older adults, from healthy to Alzheimer's demented. Regression lines were added to aid visualization, but they do not account for interdependencies within participants and covariates, unlike the model predictions plotted in the main text.
Abbreviations: MDRS, Mattis Dementia Rating Scale.

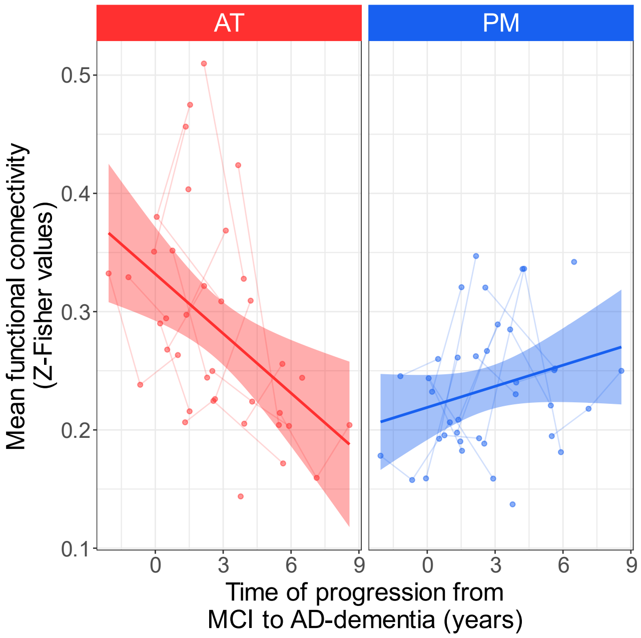

<u>Supplementary Figure 4</u>. Association between delay to Alzheimer-dementia onset and functional connectivty in the anterior-temporal (AT) and posterior-medial (PM) networks among MCI patients.
Regression lines were added to aid visualization, but they do not account for interdependencies within participants and covariates, unlike the model predictions plotted in the main text.
Abbreviations: MCI, mild cognitive impairment.

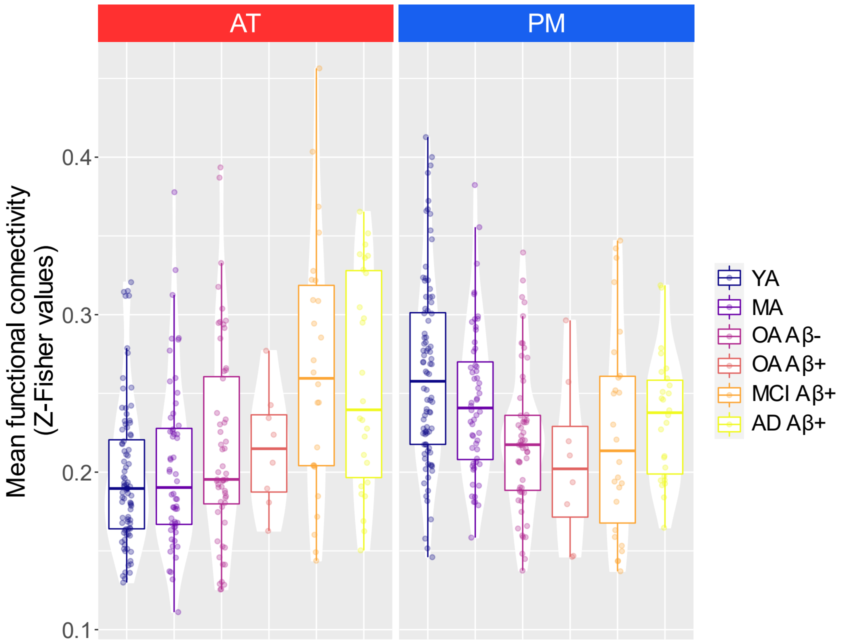

<u>Supplementary Figure 5</u>. Baseline group differences in functional connectivity within the anterior-temporal (AT) and posterior-medial (PM) networks across the whole sample.
Boxplot estimates do not account for covariates, unlike the model predictions plotted in the main text.
Abbreviations: AD: Alzheimer's disease, MA: middle-aged adults (40-60), MCI: mild cognitive impairment, OA: older adults (>60), YA: young adults (19-39).

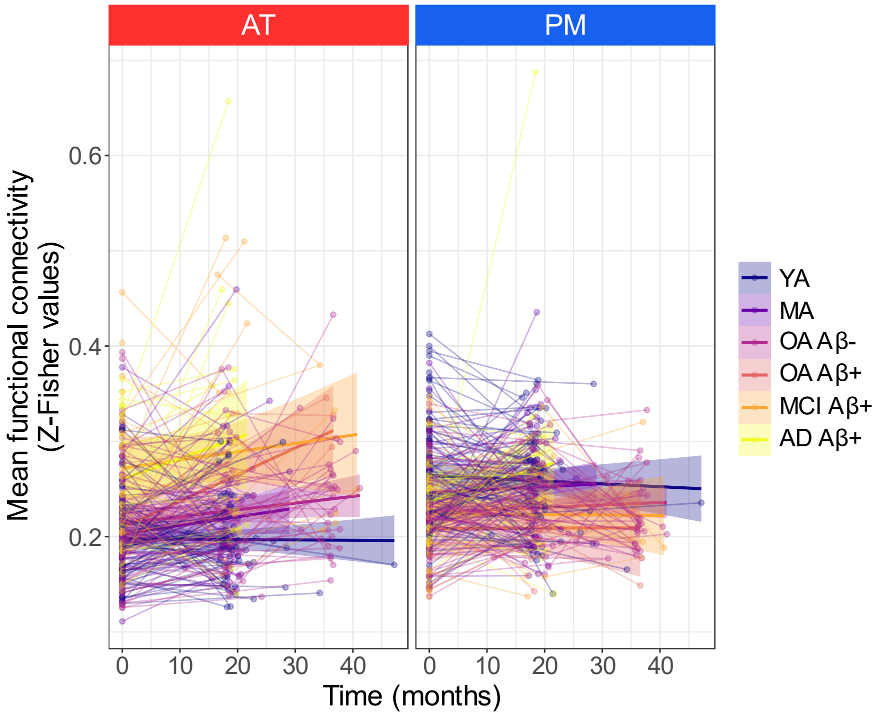

<u>Supplementary Figure 6</u>. Group differences in functional connectivity changes over time within the anterior-temporal (AT) and posterior-medial (PM) networks across the whole sample.
Regression lines were added to aid visualization, but they do not account for interdependencies within participants and covariates, unlike the model predictions plotted in the main text.
Abbreviations: AD: Alzheimer's disease, MA: middle-aged adults (40-60), MCI: mild cognitive impairment, OA: older adults (>60), YA: young adults (19-39).
